# GPCRVS - AI-driven Decision Support System for GPCR Virtual Screening

**DOI:** 10.3390/ijms26052160

**Published:** 2025-02-27

**Authors:** Dorota Latek, Khushil Prajapati, Paulina Dragan, Matthew Merski, Przemysław Osial

**Affiliations:** University of Warsaw, Faculty of Chemistry, 1 Pasteur St, 02-093 Warsaw, Polandp.dragan@uw.edu.pl (P.D.);

**Keywords:** machine learning, gradient boosting machines, neural networks, deep learning, decision support systems, drug selectivity, G protein-coupled receptors, virtual screening, molecular docking

## Abstract

G protein-coupled receptors (GPCRs) constitute the largest and most frequently used family of molecular drug targets. The simplicity of GPCR drug design results from their common seven-transmembrane-helix topology and well-understood signaling pathways. GPCRs are extremely sensitive to slight changes in the chemical structure of compounds, which allows for the reliable design of highly selective and specific drugs. Only recently has the number of GPCR structures, both in their active and inactive conformations, together with their active ligands, become sufficient to comprehensively apply machine learning in decision support systems to predict compound activity in drug design. Here, we describe GPCRVS, an efficient machine learning system for the online assessment of the compound activity against several GPCR targets, including peptide- and protein-binding GPCRs, which are the most difficult for virtual screening tasks. As a decision support system, GPCRVS evaluates compounds in terms of their activity range, the pharmacological effect they exert on the receptor, and the binding mode they could demonstrate for different types and subtypes of GPCRs. GPCRVS allows for the evaluation of compounds ranging from small molecules to short peptides provided in common chemical file formats. The results of the activity class assignment and the binding affinity prediction are provided in comparison with predictions for known active ligands of each included GPCR. Multiclass classification in GPCRVS, handling incomplete and fuzzy biological data, was validated on ChEMBL and Google Patents-retrieved data sets for class B GPCRs and chemokine CC and CXC receptors.

## 1. Introduction

The majority of the human proteome is still considered undruggable or too complex to propose therapeutics that are efficient, selective, and/or specific enough [1]. Among successful drug targets, the largest protein population belongs to ion channels, followed by G protein-coupled receptors (GPCRs), kinases, and finally nuclear receptors [1]. However, known small-molecule drugs of high bioavailability mostly target GPCRs (33%), followed by ion channels (18%), nuclear receptors (16%), and kinases (3%). A distinctive feature of GPCRs is a plethora of different types of ligands exerting varied effects on receptor activation. For example, agonists facilitate GPCR activation by stabilizing conformations that promote interactions with G proteins or other signaling proteins [2]. Antagonists prevent agonist binding but do not affect basal (agonist-independent) receptor activity, in contrast to inverse agonists [3]. Superagonists are characterized by a higher efficacy than the endogenous agonist of a receptor [4]. Allosteric modulators bind to a secondary binding site and can enhance (positive allosteric modulators) or reduce (negative allosteric modulators) the receptor’s response to an agonist [5]. Finally, ago-allosteric modulators display both receptor-modulating activity and agonist-like behavior [6].

The structural similarity of GPCRs and their common mode of activation during signal transduction imposes problems in drug discovery regarding unwanted off-target effects and a lack of drug selectivity. This requires the massive testing of drug candidates against many possible protein targets prior to clinical studies, leading to highly increased drug development costs. Recent advances in machine learning (ML) and artificial intelligence (AI) techniques demonstrated unexpected possibilities in accelerating the validation of drug candidates through the automatic detection of unwanted similarities in their chemical structures and modes of action. Mimicking human expertise in finding patterns responsible for compound activity against a certain molecular target is a major advantage of ML/AI systems currently being developed for drug design. Although such systems are often considered ‘black box’ techniques, uncovering their mechanisms of self-learning by using explainable AI (XAI) [7] provides another way of looking at biological problems that are inaccessible to human experts.

**Figure 1:**
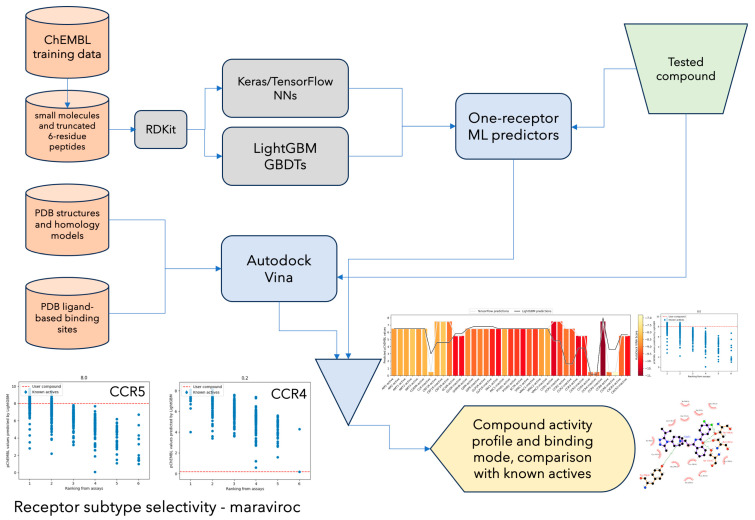
The scheme of the input data, the implemented algorithms, and the output results of GPCRVS. Bottom left—example results for maraviroc showing GPCRVS performance in terms of the receptor subtype selectivity prediction.

Here, we describe GPCRVS, a recently developed web service for virtual screening against many drug targets from class A and B GPCRs that are activated endogenously by peptides or small proteins. GPCRVS overcomes the limitations of individual methods for virtual screening, including ligand-based or target-based approaches (Supplementary Figure S1). In addition, GPCRVS allows for the testing of drug candidates in terms of their types (e.g. agonist or positive allosteric modulator, Supplementary Figure S2) and off-target effects against several well-known GPCR targets, including class B GPCRs and chemokine CC and CXC receptors (Supplementary Figures S3–S5). In particular, we propose a novel approach, six-residue peptide truncation, to combine an overwhelming number of active peptide compounds available for GPCRs with small-molecule compounds into one complete training data set (Figure 1, Supplementary Figure S6). GPCRVS was tested and compared with similar, currently accessible methods for drug design [8,9,10].

## 2. Materials and Methods

### 2.1. Deep Neural Networks Implemented in GPCRVS

Ligand-based virtual screening in GPCRVS includes two diverse ML algorithms (Figure 1): a multilayer neural network (TensorFlow version 2.15.0 [11,12]) and gradient boosting decision trees (LightGBM version 4.3.0 [13]). Keras API version 2.15.0 was used to build a sequential model of deep neural networks (DNNs) (Adaptive Moment Estimation optimizer [14], categorical cross-entropy [15]), followed by RandomSearch hyperparameter optimization (by keras_tuner [16]) and model evaluation by 10-fold cross-validation [17,18] (Supplementary Figure S7). Ten-fold cross-validation was performed using KFold from scikit-learn version 1.3.2. The best DNN model, including a first flatten layer, five to twenty hidden layers of varied activation (relu, tanh, and sigmoid) and output (16 to 2048) layers, and a final softmax layer, was implemented in GPCRVS for multiclass classification tasks (ranges of pChEMBL values [19]).

### 2.2. Gradient Boosting Machines Implemented in GPCRVS

LightGBM outperforms other gradient boosting methods due to gradient-based one-side sampling and exclusive feature bundling. It was used to build a gradient boosting machine (GBM) model with decision tree base learners and the RandomizedSearch optimization of hyperparameters (scikit-learn RandomizedSearchCV) with 10-fold cross-validation (e.g., 50 to 50,000 estimators, Supplementary Figures S8 and S9) that is comparable to the hyperparameter tuning by Optuna [20]. Ten-fold cross-validation was performed using scikit-learn (sklearn) KFold (kfold.split). The best GBM model was implemented in GPCRVS for compound activity prediction (pChEMBL values).

### 2.3. Ligand Binding Mode Prediction in GPCRVS

The flexible ligand docking mode of AutoDock Vina version 1.2.3 [21], previously extensively tested against several class B GPCRs [22] in comparison with the Schrödinger-licensed Glide [22,23] and successfully used in [24], was implemented in GPCRVS (Supplementary Figures S10 and S11). Orthosteric and allosteric binding sites (including different sites for known PAMs and NAMs) were considered separately with the PDB ligand-based centering of the 30 Å cubic search box. Receptor structures were either based on PDB structures with Maestro-based missing atom filling and refinement [25] or, if missing, prepared with the previously described Modeller/Rosetta CCD loop modeling approach to GPCR structure prediction [24] implemented in GPCRM [26,27].

### 2.4. Data Sets Used for Training and Validation

Data sets (Supplementary Figure S1) were retrieved from ChEMBL (accessed before June 2024) and curated in terms of uniqueness (the removal of duplicates), SMILES correctness (the ability to transform SMILES into a molecule), and assay type compatibility (assay type ‘F’, denoting a functional assay). This was achieved using RDKit version 2023.09.2. An 80/20% ratio between training and validation sets was applied with random splitting. Peptide compounds, many consisting of nearly 30 amino acids, were truncated to the 6-residue-long N-terminal fragments, the most relevant for the receptor activation, and converted to SMILES. Peptide compounds activating opioid, chemokine, and class B receptors tend to bind with their N-terminal short fragments (or close to the N-terminus [28]), which carry the activation ‘message’ [29,30,31], while C-terminal regions (‘addresses’) are responsible for the correct orientation, binding stability, and localizing interactions with the receptor and the ECD domain and/or lipid bilayer preceding the receptor binding [32]. Certain other receptors, not included in GPCRVS, such as apelin, endothelin, or neurotensin receptors, have C-terminal activating fragments instead [33,34]. To retrieve compound-unique chemical features, extended connectivity fingerprints with a bond diameter equal to 4 (ECFP4) based on Morgan fingerprints were used [35,36]. In molecular docking, these short peptides were treated as small molecules, with flexibility limited by the Autdock Vina flexible ligand docking mode and 3D conformation generation with OpenBabel version 3.1.1.

The curated ChEMBL-retrieved data sets (Supplementary Figure S6) were used as training and testing sets (80% and 20%, respectively) to evaluate the method’s performance (Supplementary Figures S3 and S4). In addition, two validation data sets were used to evaluate the method. The first data set, with three highly active compounds for each GPCR, was retrieved from ChEMBL and manually checked for target selectivity, SMILES format correctness for fingerprint generation (RDKit, Chem.MolFromSmiles from the rdkit.Chem.rdmolfiles module, described in detail in [36]), file format conversion (smi, pdb, pdbqt) by AutoDock MGLTools version 1.5.7, 3D coordinate generation from SMILES (OpenBabel), partial charges assignment (AutoDock MGLTools), etc. Notably, many of the compounds removed from this original ChEMBL data set failed at the 3D coordinates generation step, providing several receptor subtype-selective compounds but also many non-selective compounds (Supplementary Table S1). The second data set included 140 patent compounds obtained from Google Patents for CCR1, CCR2, CCR6, CXCR2, CXCR3 (inhibitors, class A), CRF1R (inhibitors, class B), GCGR, GLP1R, GIPR, and VPAC2 (agonists, class B). This data set also included several peptide compounds that were truncated to 6-residue fragments before evaluation. Both Google Patents and SwissTargetPrediction were accessed in November 2024.

### 2.5. Third-Party Software Used in GPCRVS and Update Procedures

Open-source, third-party software and frameworks that were used in GPCRVS include TensorFlow 2.15.0, Keras API 2.15.0, LightGBM 4.3.0, AutoDock Vina 1.2.3, MGLTools 1.5.7, Open Babel 3.1.1, RDKit 2023.09.2, scikit-learn 1.3.2, Pandas 2.0.1, Three.js r157, JSME 2023-07-31, React 18.3.1, Django 4.2, MySQL 8.0.32, Apache 2.4.53, and Rocky Linux 9.2. Compound training data sets were retrieved from ChEMBL 34. GPCRVS was tested on Google Chrome, Safari, Mozilla Firefox, and Edge, and on CentOS Stream, Rocky Linux, Ubuntu, Windows 11, and macOS. Currently, GPCRVS includes 39 receptor structures (26 unique receptor types). Additional python scripts ensure its automatic update in conjunction with increasing PDB and ChEMBL database resources. These include the preparation of new receptor models/structures, the retrieval and curation of new ligand data sets, training with data sets for new receptors or retraining with updated data sets for already included receptors, new ML model generation and updates, and the addition of new ligand activity predictions to the comparison data sets provided for each receptor.

## 3. Results

### 3.1. GPCRVS as a Decision Support System for Newly Designed Compounds

The typical output provided by GPCRVS includes a compound profile representing its predicted activity against several GPCR drug targets (Figure 1). There are three components of this decision support system—a DNN-based classification of compound activity to one of six activity classes, a GBM-based prediction of compound activity, and an AutoDock Vina-based prediction of compound binding affinity. The former two components rely on the pChEMBL normalization of compound activity markers such as IC50. Compound binding affinity prediction is based on a scoring function implemented in AutoDock Vina, including only van der Waals-like potential, hydrogen bonding, and hydrophobic terms, as well as a conformational entropy penalty, in contrast to the much less computationally efficient AMBER-based force field of AutoDock4.2 involving electrostatics [21]. In addition to their activity and off-target prediction, compounds are positioned in every orthosteric/allosteric binding site of all of the receptors with advanced AutoDock Vina sampling of the ligand–protein conformational space using a Monte Carlo search algorithm that includes an improved implementation of macrocycle flexibility [21]. The performance of AutoDock Vina in binding mode prediction was previously extensively tested [37], as well as with GPCR ligands [38,39], and proved to be slightly better in the prediction of binding poses than AutoDock4 [37].

These three components of the GPCRVS decision support system represent completely different approaches to virtual screening (ligand-based vs. target-based, DNN-based vs. GBM-based, multiclass classification vs. regression). Thus, the compound activity prediction is not biased by an overlap between component methods but is solely due to the compound structure and resulting properties. Assuming that only a single predictor is needed to succeed in the prediction task, the accuracy of GPCRVS was 71.4% (GPCR-like, selective data set, Supplementary Table S3). If two of three predictors are needed to predict the correct receptor type, the accuracy was 33.3% (28.6% if only the ML predictors were considered). Although the resulting average prediction accuracy for the whole blind test set, including non-selective ligands, seems low (52.4%), it is still much higher than the prediction accuracy of each of the methods separately (28.6%, 26.2%, and 16.7% for the DNNs, GBMs, and AutoDock Vina, respectively); thus, a significant improvement is observed compared to our previous AutoDock Vina-based web service [23]. Moreover, it exceeds the prediction accuracy of similar methods (31.0% for the best method, Supplementary Table S2) and thus offers the low-cost pre-testing of drug candidates, which is unachievable using typically costly screening with pharmacological assays that are effective against a few GPCR targets at most [40,41].

GPCRVS demonstrated a high prediction accuracy for the validation of GPCR ligand data sets, which are added for comparison to the user’s predictions (Supplementary Figures S1–S5). Namely, for most of the included GPCRs, the compound activity ranking (provided as pChEMBL values) based on ChEMBL data from functional assays correlated with the DNN/GBM/AutoDock Vina predictions (Supplementary Figure S1). GPCRVS also performed well at binary classification, involving predictions for negative data points (pChEMBL equal to 0), as tested previously in [19]. This showed its applicability in binary active/inactive ligand classification tasks.

### 3.2. Decoding the Feature Importance in LightGBM

**Figure 2:**
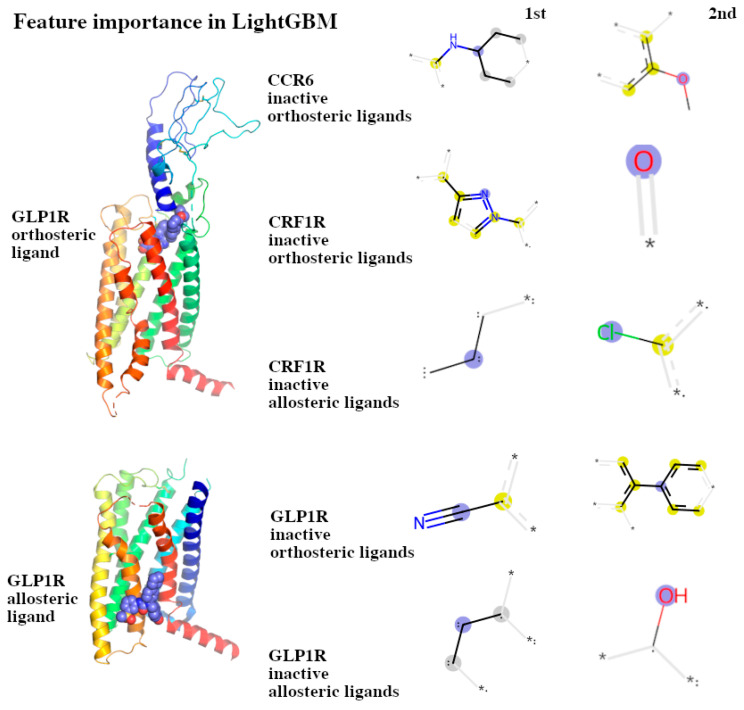
Examples of the feature importance obtained for the CCR6, CRF1R, and GLP1R orthosteric and allosteric ligand training sets. Here, the 1st and 2nd most important structural features for each training set were presented. The most important structural features (fingerprint bits) were selected using the feature importance gain method in LightGBM and drawn with RDKit. Yellow atoms are aromatic atoms, blue dots represent the centers of the fragment, and other atoms and bonds are marked with grey with the continuation of the fragment marked with asterisks.

To decode feature importance in the training data sets, we used the feature importance gain method implemented in LightGBM (Figure 2 and Figures S12–S16). This explanation of the compound structural features presented below, which were the basis for performing regression tasks using GBDTs implemented in LightGBM, clearly shows that the training sets used for each receptor are inherently different in terms of the resulting feature importance. What is more, LightGBM seems to be sensitive to the hydrophobic/hydrophilic properties of compounds. For example, the orthosteric ligands of GLP1R bind to the receptor at the cytoplasm-facing, extracellular side and include hydrophilic fragments, while allosteric ligands bind to the receptor within the lipid bilayer and have hydrophobic properties. This is indeed observed in the feature importance detected by the gain method in LightGBM. The most important feature of GLP1R orthosteric ligands includes a structural fragment that could be involved in forming hydrogen bonds, while the most important feature of GLP1R allosteric ligands includes only an aliphatic carbon chain that could interact with the hydrophobic tails of cell membrane phospholipids.

### 3.3. Comparison with Similar Methods

**Figure 3:**
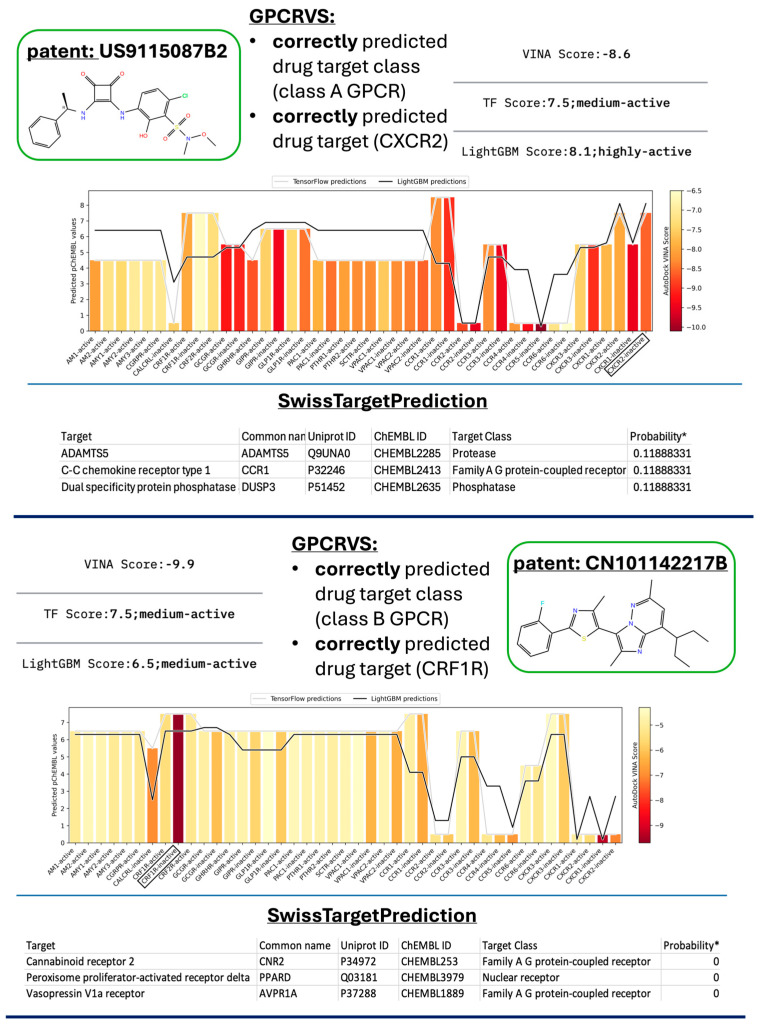
Example results for two patent compounds for which GPCRVS correctly predicted both the drug target and drug target class. Asterisks - the probability score provided by SwissTargetPrediction mean: ‘probability for the query molecule—assumed as bioactive—to have this protein as a target’.

To our knowledge, there is no web service with all three of the modules that are currently available in GPCRVS. Nevertheless, some of these methods, especially those based on machine learning, are available in web services for target prediction. For example, CODD-Pred [8], SuperPred [9], and SwissTargetPrediction [10] were not developed specifically for GPCRs but for all known drug targets, mostly including three drug target classes other than GPCRs: ion channels, nuclear receptors, and kinases (estimation performed in 2017 by Santos et al. for small-molecule drugs [1]). In Supplementary Table S2, we provided a comparison of the recent performance (June 2024) of GPCRVS with the SwissTargetPrediction web service in target/target class prediction for a GPCR-like validation data set. Large databases of compounds such as ChEMBL, PubChem, or ZINC15 were not included in the comparison, as their data sets were included in the training sets. The top three target predictions provided by SwissTargetPrediction (but only one for GPCRVS) were considered. In Supplementary Table S3, we provided the rates of true positives for these two methods. A ‘true positive’ refers to the correct assignment of a compound to either a GPCR type or to GPCR-like ligands regardless of the receptor type. The performance of GPCRVS was comparable with SwissTargetPrediction. Interestingly, AutoDock Vina outperformed TF and LightGBM in the target class prediction, while it performed worse in the more precise target subtype prediction. This shows the ability of AutoDock Vina to distinguish between diverse binding sites of class A and class B GPCRs [22,23,38], but also its limitations in distinguishing subtle structural differences between receptor subtypes (e.g., serotonin receptor subtypes [24]). LightGBM slightly outperformed TF due to being better fitted to regression tasks, such as the prediction of the pChEMBL value of the compound [19,38]. Similar results were obtained for the patent compound data set retrieved from Google Patents (Table 1, Supplementary Tables S4–S13). An example of the results obtained for two of the tested patent compounds is presented in Figure 3. Evaluation with this data set again showed that LightGBM outperformed TF. However, a thorough evaluation, e.g., GPCR Dock 2013 [24], that includes compounds deposited neither in ChEMBL nor PubChem is certainly needed to reliably compare the aforementioned methods.

Although this evaluation could certainly be extended by including non-GPCR ligands, e.g., kinase ligand data sets, it shows that GPCRVS is a highly accurate tool for searching for GPCR ligands when compared to the currently available methods also developed for other drug target classes. In particular, the target class assignment (class A vs. class B in the case of GPCRVS, and GPCR vs. non-GPCR in the case of others) is more accurate compared to the other currently accessible (June and November 2024) tested methods. This noticeably higher performance of GPCRVS is in spite of using two different data sets of GPCR ligands derived from ChEMBL and from Google Patents. Although it is only a first attempt to a more reliable comparison, including e.g. non-GPCR ligands, GPCRVS clearly showed its high performance as a decision support system. Notably, the main aim of GPCRVS is not to assign a target class to a tested compound from among all possible drug target classes, including non-GPCR-like classes, as there are already many such methods [8,9,10], not to mention ChEMBL itself. The added value of GPCRVS is providing three, diverse in principle, algorithms that indeed provide internally uncorrelated results (Table 1, Supplementary Tables S2 and S3, Figures S1, S3 and S4), to assess compound selectivity, affinity, and activity range for GPCR targets.

## 4. Conclusions

GPCRVS represents an efficient, simple, easily accessible, and open-source web service that, as a decision support system, aims to facilitate the preclinical testing of drug candidates targeting peptides and small protein-binding G protein-coupled receptors. There are three major areas of drug discovery that GPCRVS could facilitate: prediction of drug selectivity, prediction of drug efficacy approximated by Autodock Vina docking scores, or by activity class assigned by the TensorFlow multiclass classifier, or by pChEMBL predictions using the LightGBM regressor, and finally prediction of the drug binding mode, showing the most crucial amino acids involved in the drug-receptor interactions. Not only is drug target selectivity considered, but due to the implementation of receptors from two GPCR classes (A and B), drug target class selectivity is also taken into consideration. This is especially important for testing peptide compounds. A comparison with precomputed results for known active compounds enables the prioritization of drug candidates, thereby significantly reducing the cost and length of experimental screening.

In addition, a novel approach to using peptide ligand data sets as SMILES-based fingerprints in conjunction with small-molecule ligand data sets in the training of DNN and GBM models was proposed. This six-residue truncation of peptides to their most relevant fragments makes it possible to benefit from all GPCR-like ligand data sets deposited, e.g., in ChEMBL, for every GPCR receptor and to design new drugs that could include both peptide and non-peptide scaffolds of increased, unified activity and selectivity. Currently, two groups of peptide/small protein-binding GPCR receptors are included in GPCRVS, allowing it to make comparative predictions for class A and B receptors at the same time. The evaluation of GPCRVS performed using the patent compound data set showed that LightGBM provides the most accurate results among the three predictors implemented in GPCRVS. The lack of curated data sets with a large number of active compounds for some GPCR receptors is still a major limitation for machine learning. Nevertheless, mixed training data sets, such as these used in GPCRVS for AM, AMY, GHRHR, and SCTR receptors (Supplementary Tables S14–S17), could be a temporary solution until compound databases such as ChEMBL are enriched with new experimental data from functional assays.

## Figures and Tables

**Table 1:** Results for 140 patent compounds excluded from the GPCRVS and SwissTargetPrediction training sets (accession date: 1 November 2024).

Method	Drug Target Prediction *		Drug Target Class Prediction *	
	Orthosteric **	Allosteric	Orthosteric	Allosteric
GPCRVS—all three classifiers	0.007	0.021	0.121	0.121
GPCRVS—at least 2 classifiers	0.207	0.171	0.407	0.393
GPCRVS—at least 1 classifier	0.321	0.250	0.357	0.421
GPCRVS—TF	0.229	0.271	0.464	0.493
GPCRVS—LightGBM ***	**0.450**	**0.293**	**0.643**	**0.643**
GPCRVS—Autodock Vina	0.079	0.093	0.429	0.436
SwissTargetPrediction	0.243		0.357	

* Here, precision = TP/TP + FP was computed, where TP—true positives (compounds with a correctly assigned target or target class), FP—false positives (compounds with a falsely assigned target or target class). ** Here, results for the orthosteric and allosteric ligand modes, respectively, were provided. *** Here, the best predictor and the best result were bolded.

## Data Availability

GPCRVS is freely available on the web at https://gpcrvs.chem.uw.edu.pl without registration. GPCRVS is available at: https://gpcrvs.chem.uw.edu.pl without any registration. Additional tables and figures not included in the main manuscript—S1.pdf (File S1). ChEMBL data sets (30 June 2024) used for training—S2.zip (File S2, data sets used for comparison (known active ligands)—S3.zip (File S3). GPCRVS results for patent compounds not included in training sets—S4.zip (File S4), SwissTargetPrediction (30 November 2024) results for patent compounds not included in its database—S5.zip (File S5). Patent compounds were downloaded from the Google Patents database (30 November 2024).

## Supplementary Materials

The following supporting information can be downloaded at: https://www.mdpi.com/article/10.3390/ijms26052160/s1.
