# Supplementary material for: GPCRVS - AI-driven Decision Support System for GPCR Virtual Screening
Source: Int J Mol Sci. 2025 Feb 27;26(5):2160. doi: 10.3390/ijms26052160 (PMC11900134; doi:10.3390/ijms26052160)
Supplement: Supplementary file 1 [file ijms-26-02160-s001.zip › supplementary/S1.pdf]

Supplementary

GPCRVS - AI-driven decision support system for GPCR virtual screening

Dorota Latek*, Khushil Prajapati, Paulina Dragan, Matthew Merski, Przemysław Osial

Faculty of Chemistry, University of Warsaw, Pasteur 1, 02-093 Warsaw, Poland

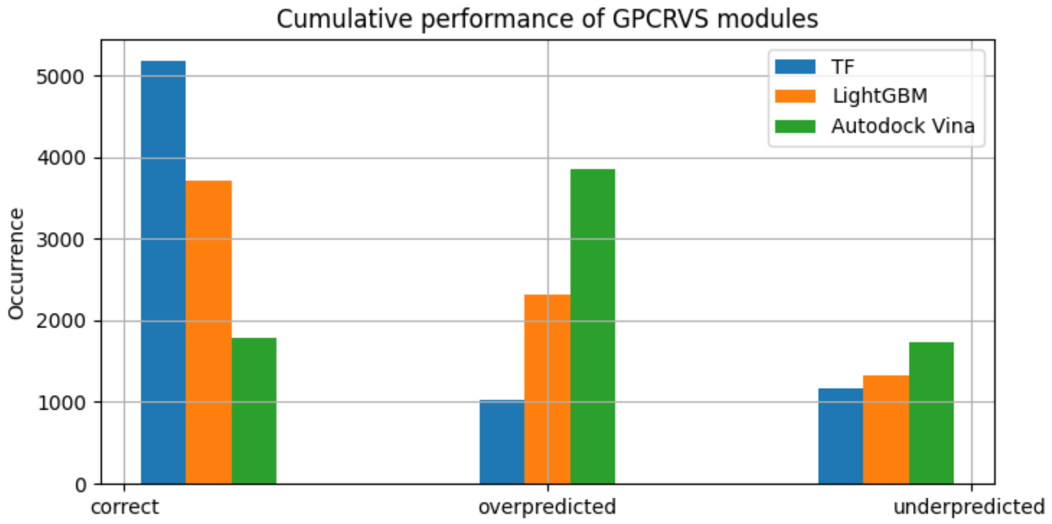

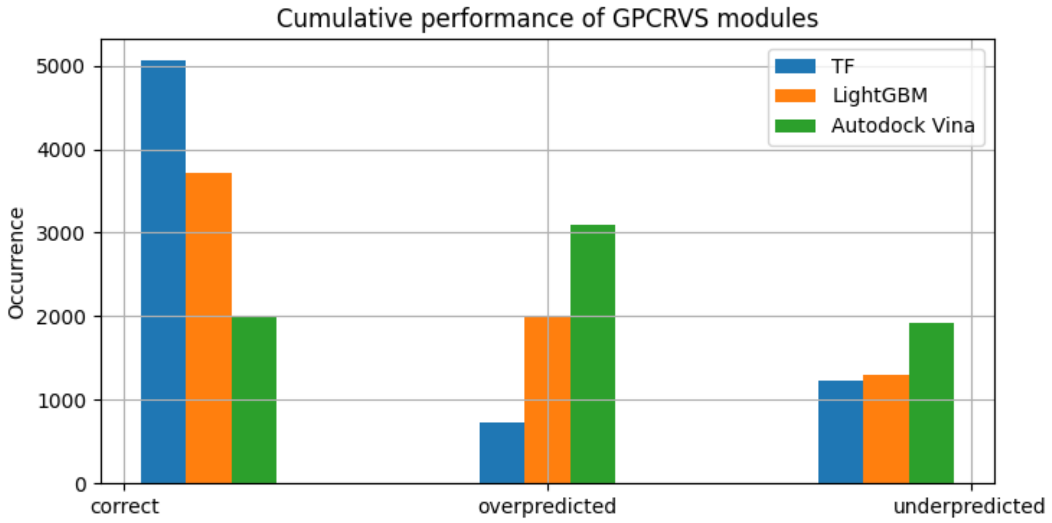

Fig. S1. The cumulative performance of the GPCRVS modules – results for ChEMBL GPCR orthosteric (top) and allosteric ligands (bottom). Here, correctness means the correct assignment of a ligand to one of six activity classes defined by pChEMBL values based on functional assays: 6 (pChEMBL < 4), 5 (4–5), 4 (5–6), 3 (6–7), 2 (7–8), 1 (pChEMBL>8). For AutoDock Vina, the ranges were obtained based on histograms obtained for the docking scores of known active ligands (Fig. S2) and were different for allosteric and orthosteric ligands. For allosteric ligands: 6 (Vina score > -3), 5 (-5 to -3), 4 (-6.5 to -5), 3 (-7.5 to -6.5), 2 (-8.5 to -7.5), 1 (Vina score < -8.5). For orthosteric ligands: 6 (Vina score > -4), 5 (-6 to -4), 4 (-8 to -6), 3 (-11 to -8), 2 (-12, -11), 1 (Vina score < -12).

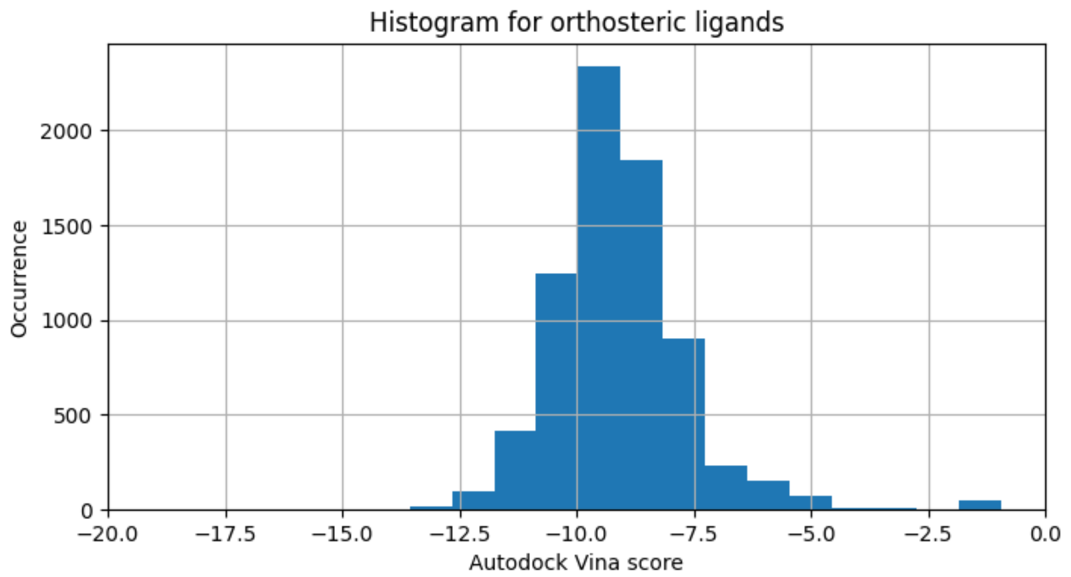

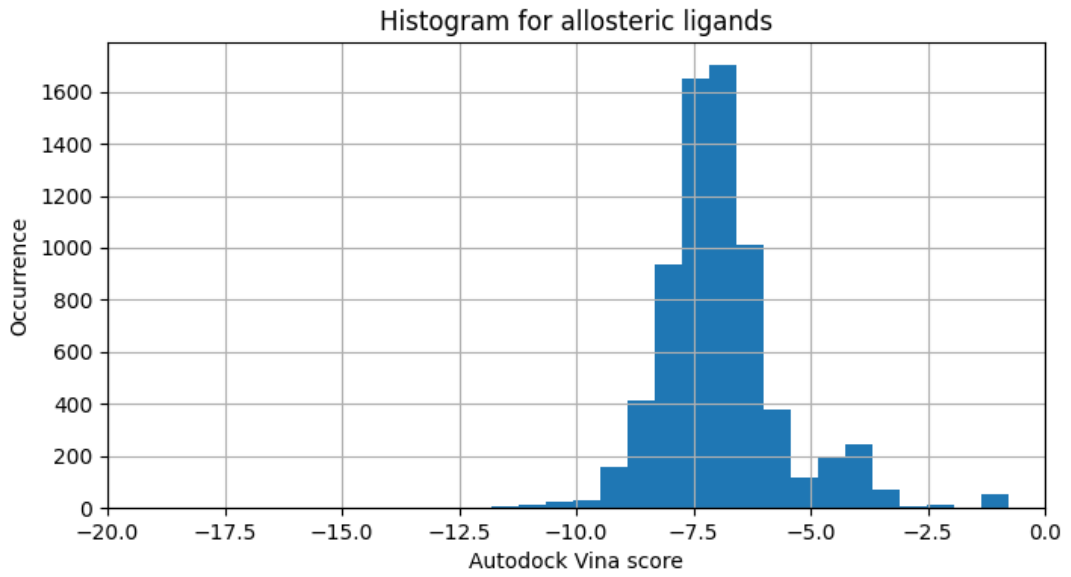

Fig. S2. Histograms obtained from the molecular docking results with AutoDock Vina for ChEMBL GPCR ligands.

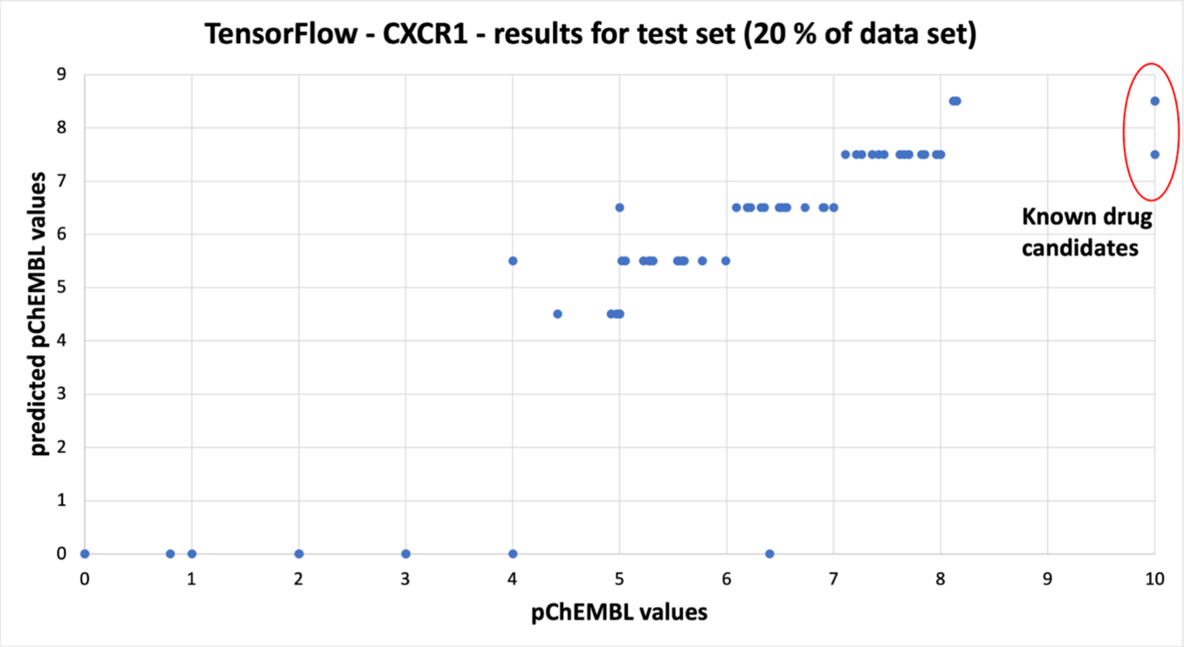

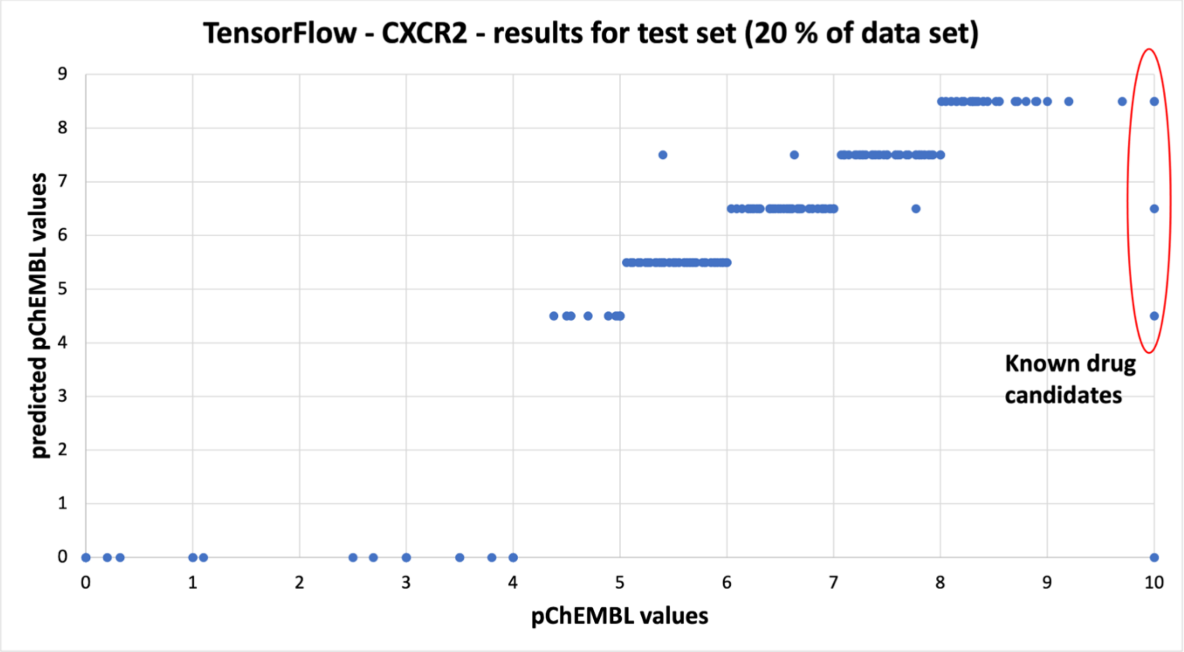

Fig. S3. The results of DNNs implemented in GPCRVS for CXCR1 and CXCR2 ChEMBL data sets. The predictions for known drug candidates in clinical phases or approved were marked with red circles.

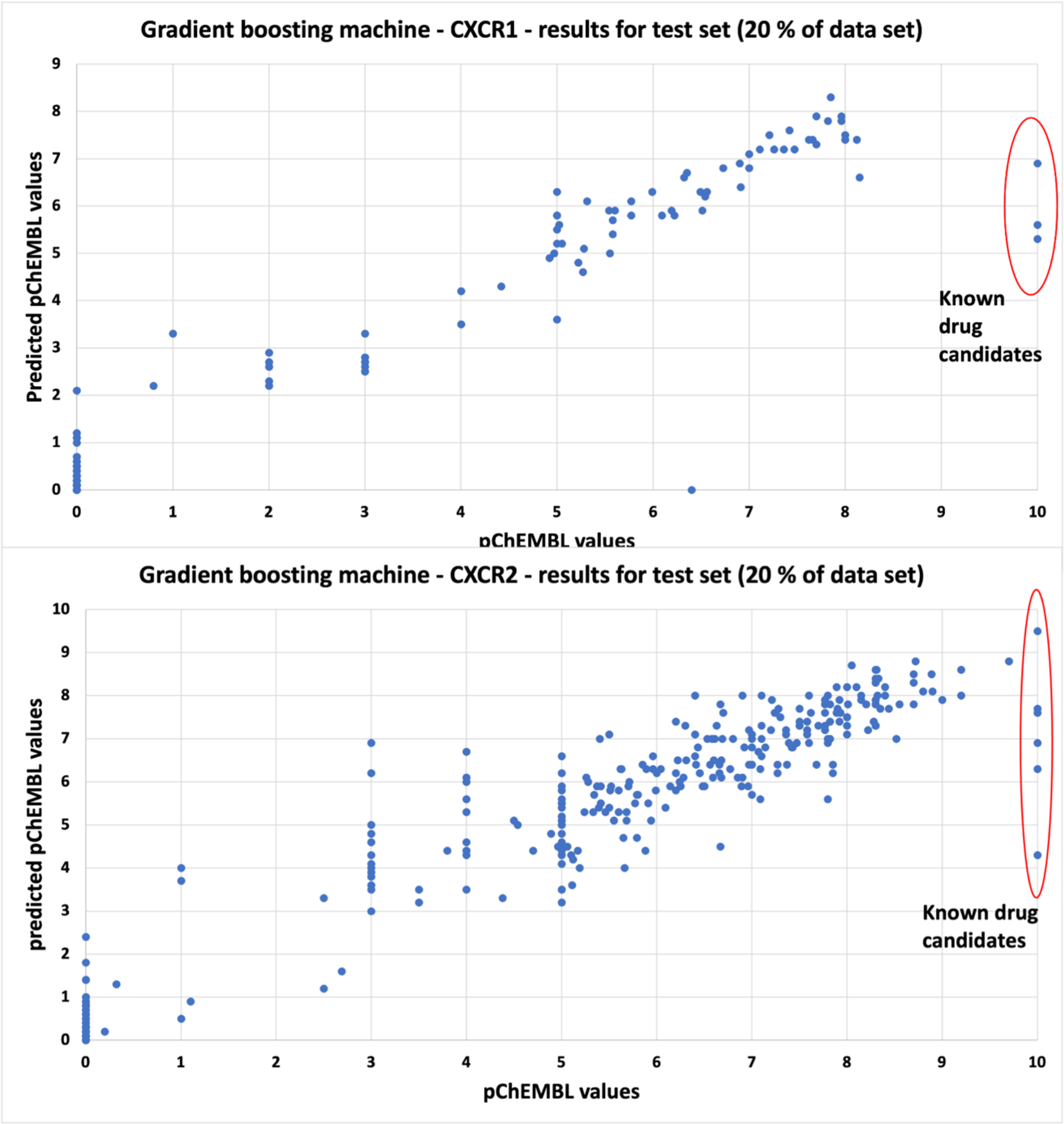

Fig. S4. The results of GBMs implemented in GPCRVS for CXCR1 and CXCR2 ChEMBL data sets. The predictions for known drug candidates in clinical phases or approved were marked with red circles.

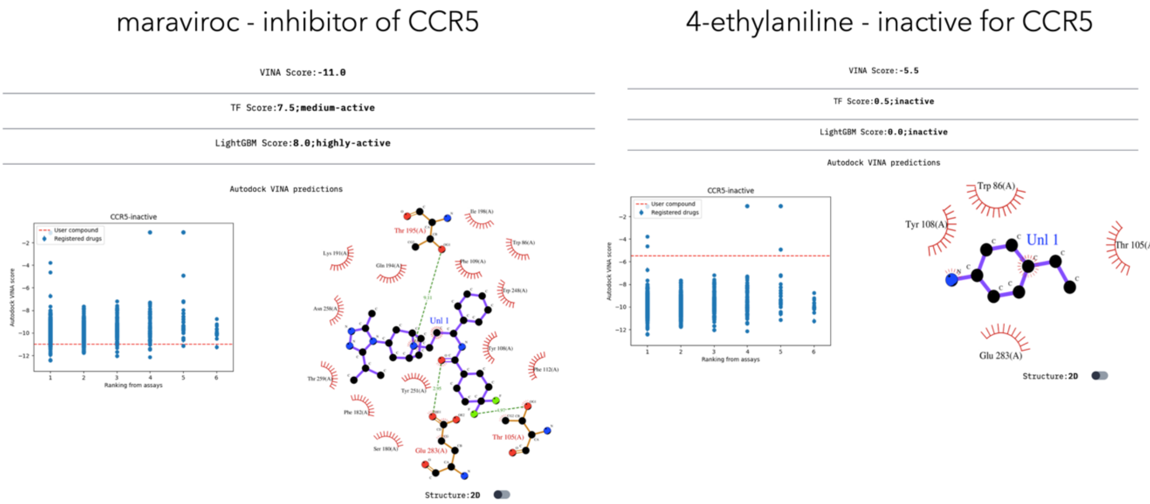

Fig. S5. Example detailed results for two diverse compounds against inactive-state CCR5.

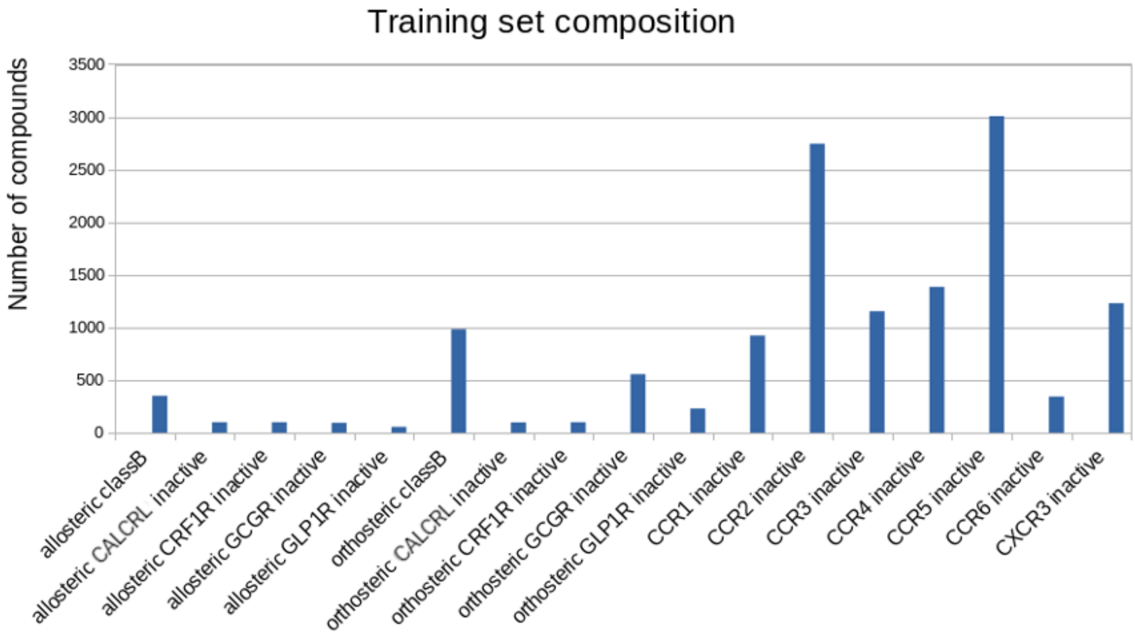

Fig. S6. The composition of ML training data sets retrieved from ChEMBL and curated – June 2024.

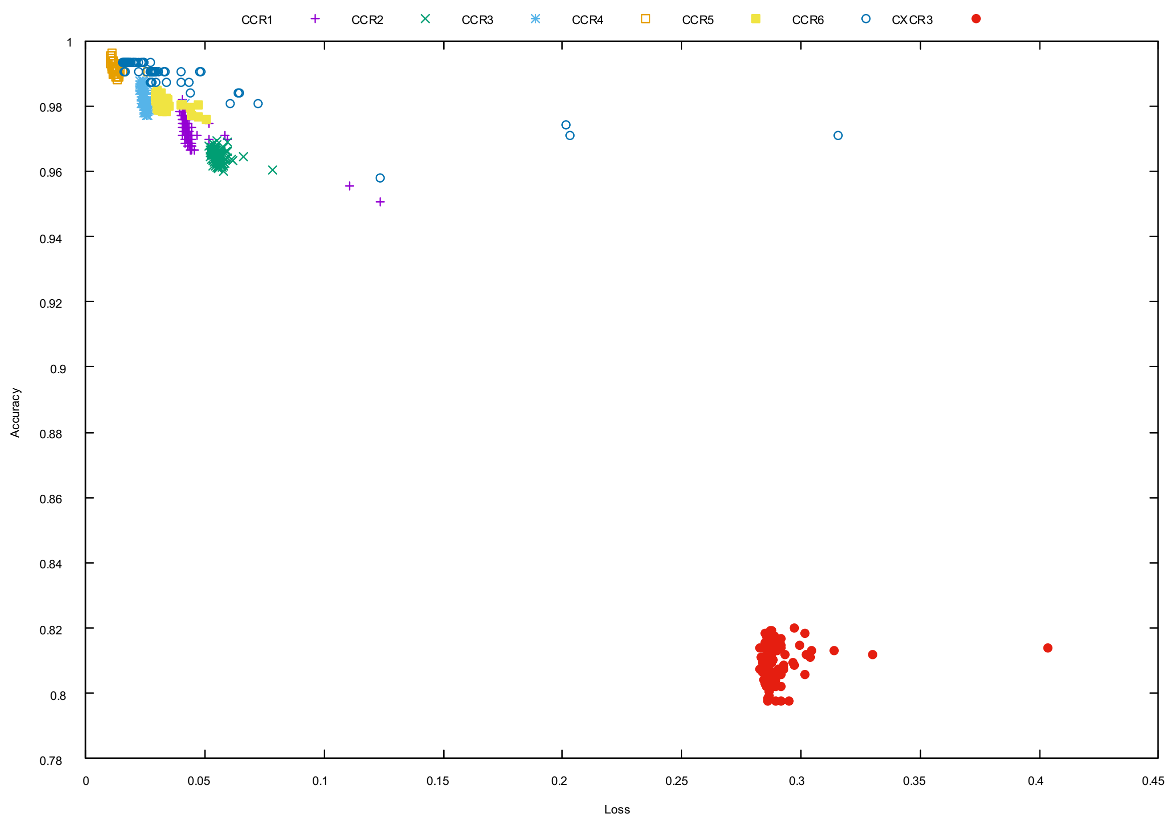

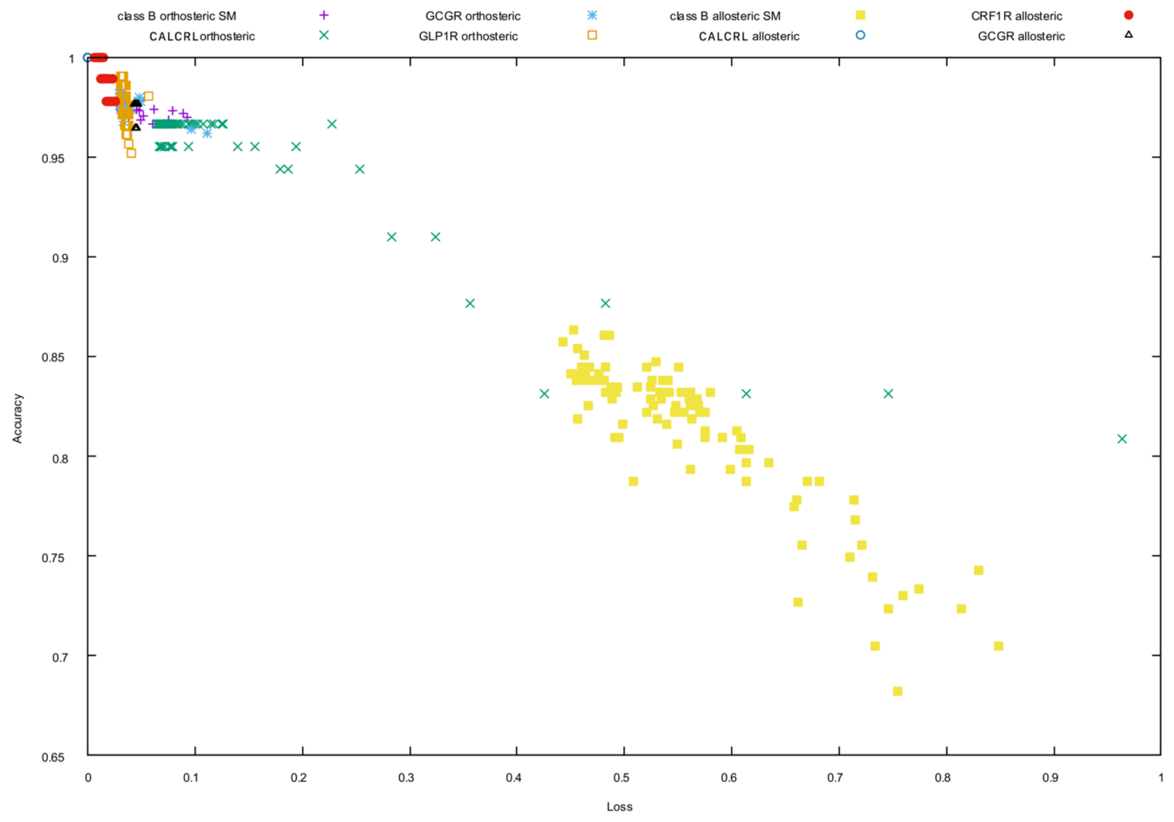

Fig. S7. Training efficiency in 10-fold cross validation tests for chemokine (top) and class B GPCR receptors (bottom) – Keras/TensorFlow deep neural networks. Each data point corresponds to an average result of one iteration of hyperparameter optimization.

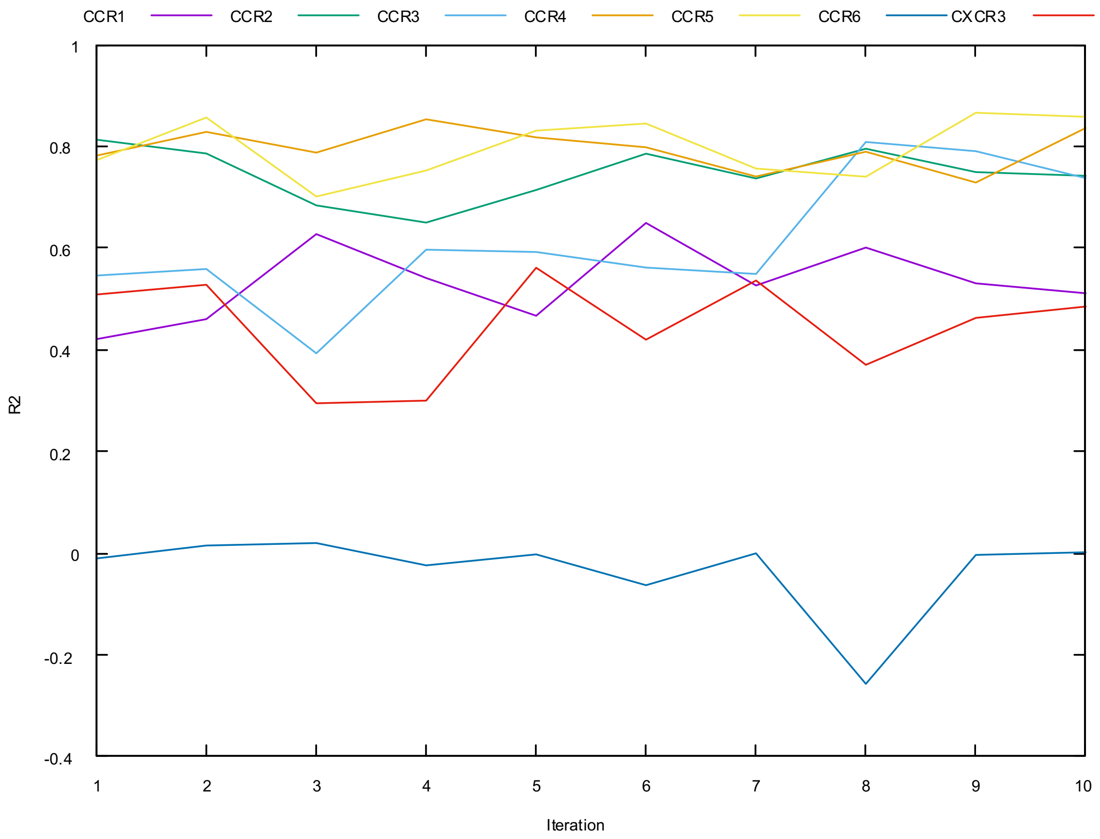

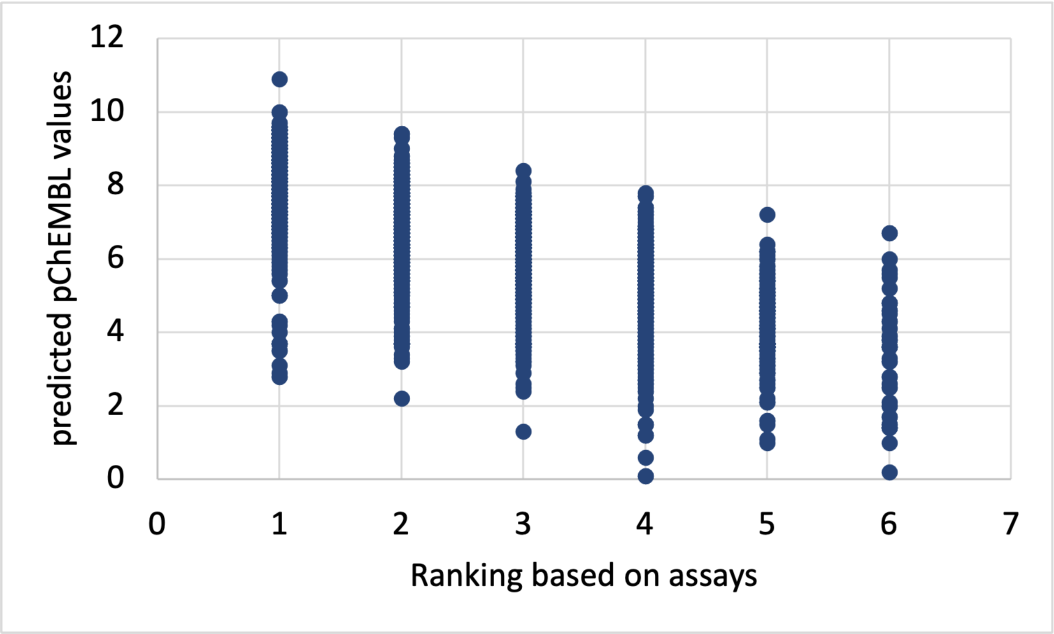

Fig. S8. Training efficiency in 10-fold cross validation tests for chemokine receptors – LightGBM gradient boosting machines with decision trees base-learners.

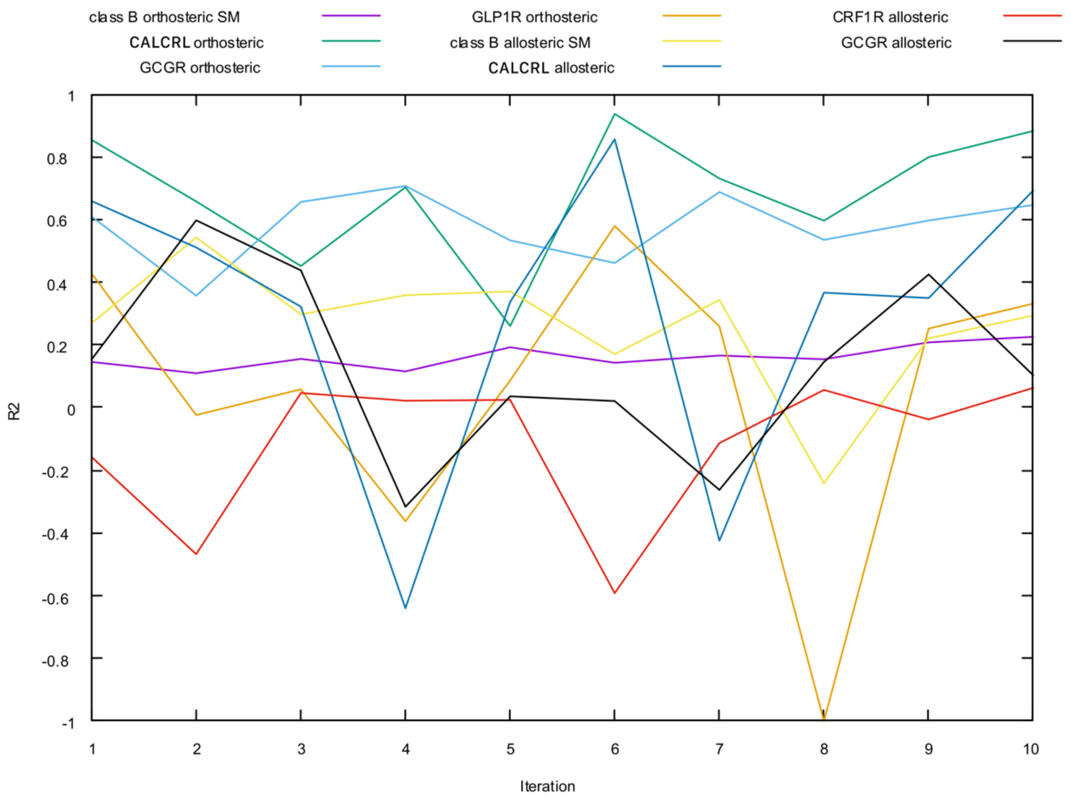

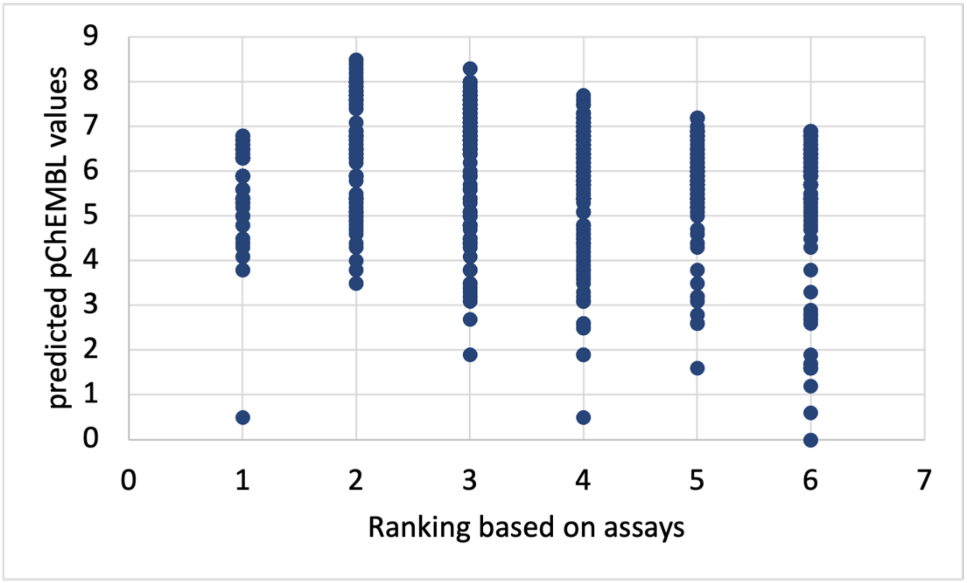

Fig. S9. Training efficiency in 10-fold cross validation tests for class B receptors – LightGBM gradient boosting machines with decision trees base-learners.

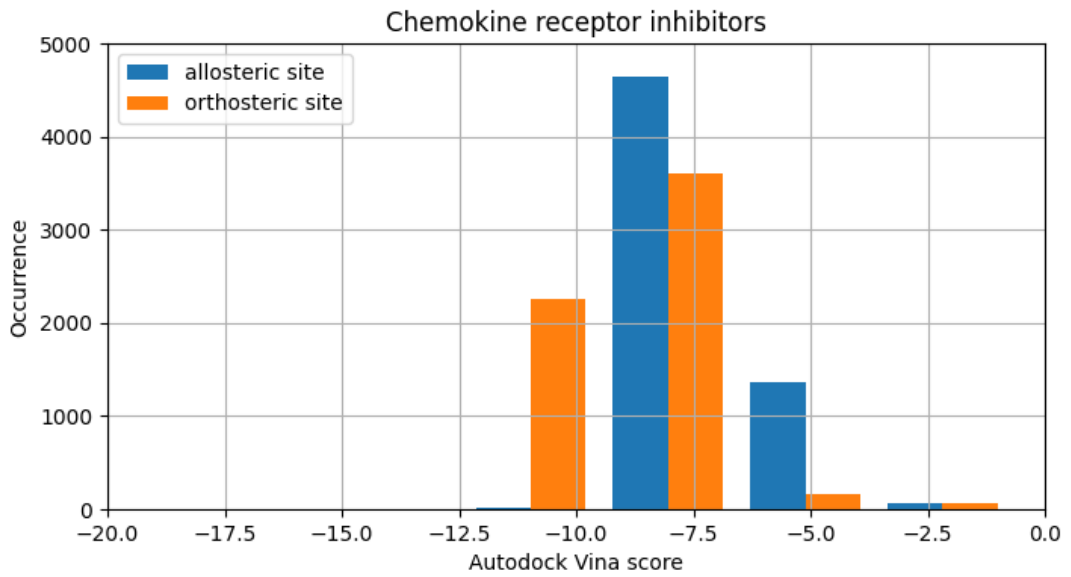

Fig. S10. The results of AutoDock Vina for the ChEMBL data set – chemokine receptor inhibitors.

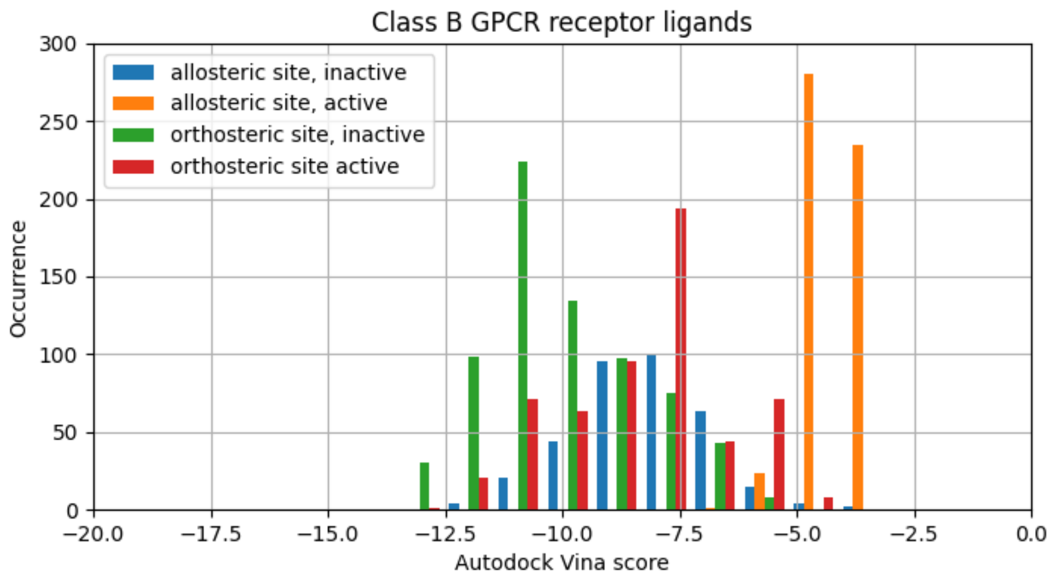

Fig. S11. The results of AutoDock Vina for the ChEMBL data set – class B GPCR ligands.

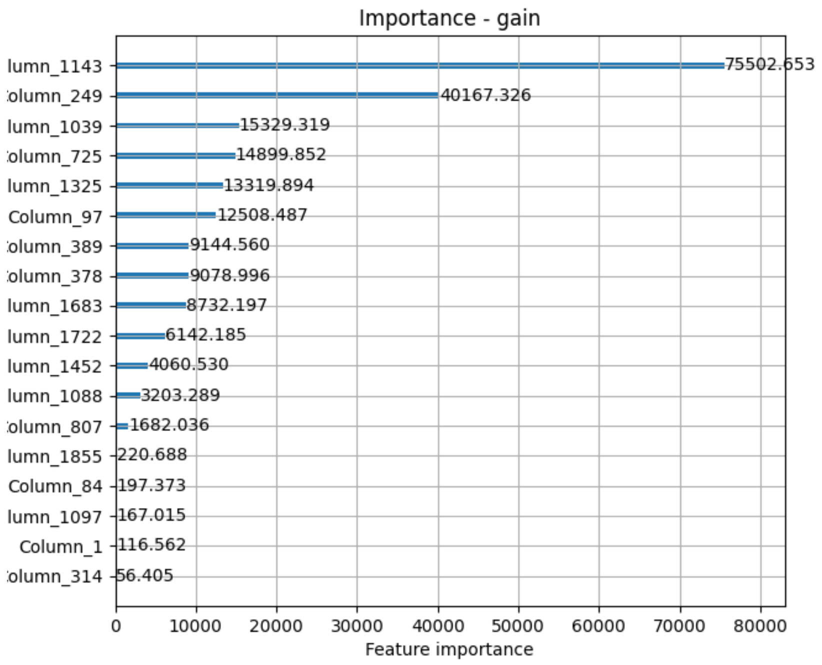

Fig. S12. Feature importance for the CCR6 inhibitors shown by the feature importance gain method in LightGBM. Here, the most important feature (depicted as column_1143) corresponds to the fingerprint bit referring to the first structural fragment shown in Figure 2, while the second structural fragment shown in Figure 2 corresponds to column_249.

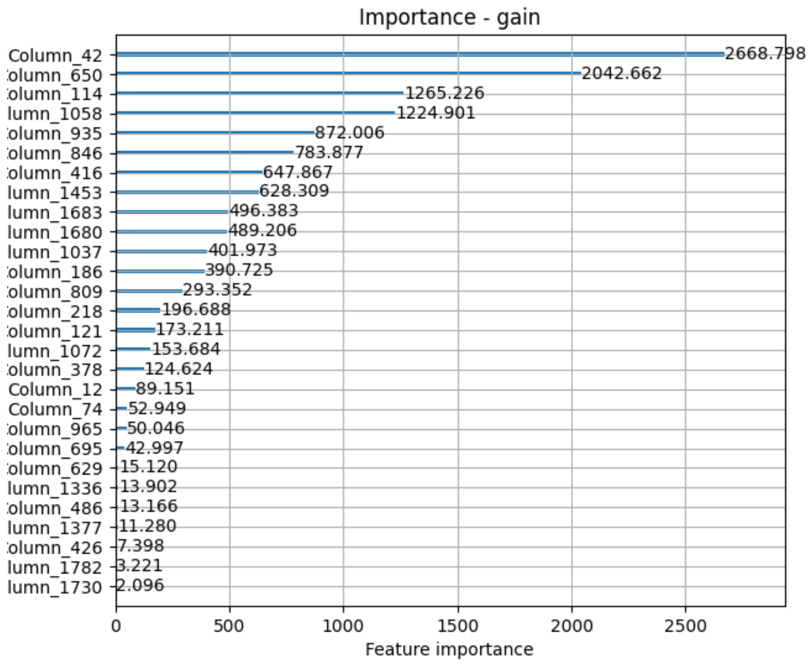

Fig. S13. Feature importance for the CRF1R orthosteric ligand data set shown by the feature importance gain method in LightGBM. Here, the most important feature (depicted as column_42) corresponds to the fingerprint bit referring to the first structural fragment shown in Figure 2, while the second structural fragment shown in Figure 2 corresponds to column_650.

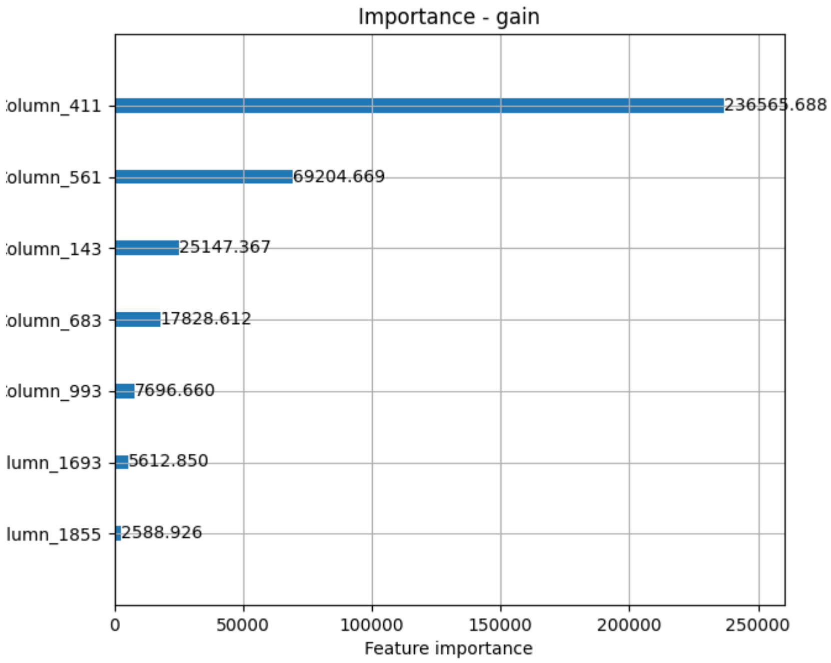

Fig. S14. Feature importance for the CRF1R allosteric ligand data set shown by the feature importance gain method in LightGBM. Here, the most important feature (depicted as column_411) corresponds to the fingerprint bit referring to the first structural fragment shown in Figure 2, while the second structural fragment shown in Figure 2 corresponds to column_561.

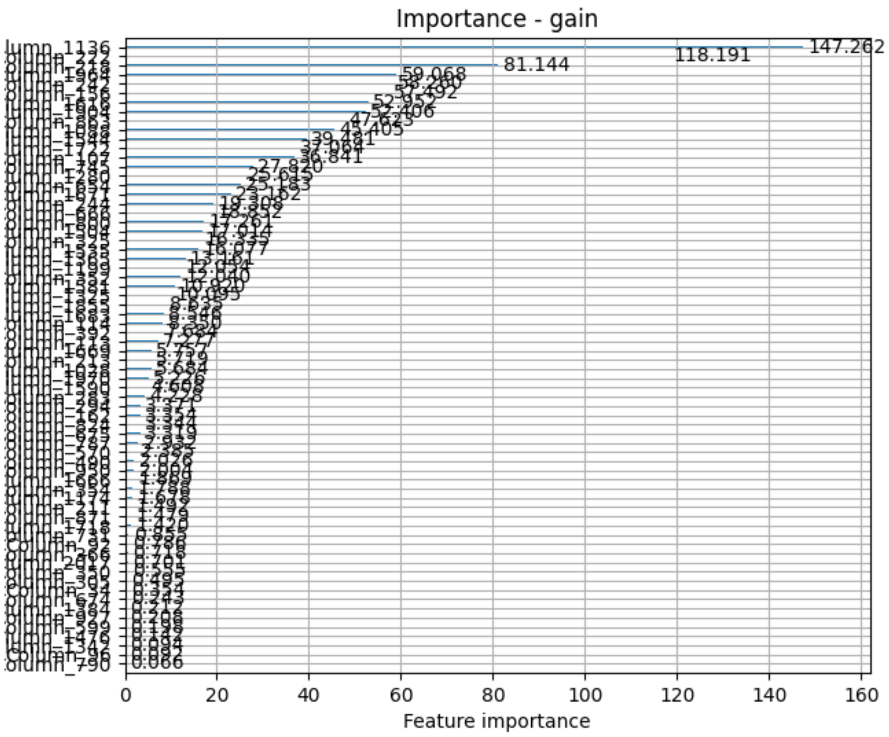

Fig. S15. Feature importance for the GLP1R orthosteric ligand data set shown by the feature importance gain method in LightGBM. Here, the most important feature (depicted as column_1136) corresponds to the fingerprint bit referring to the first structural fragment shown in Figure 2, while the second structural fragment shown in Figure 2 corresponds to column_222.

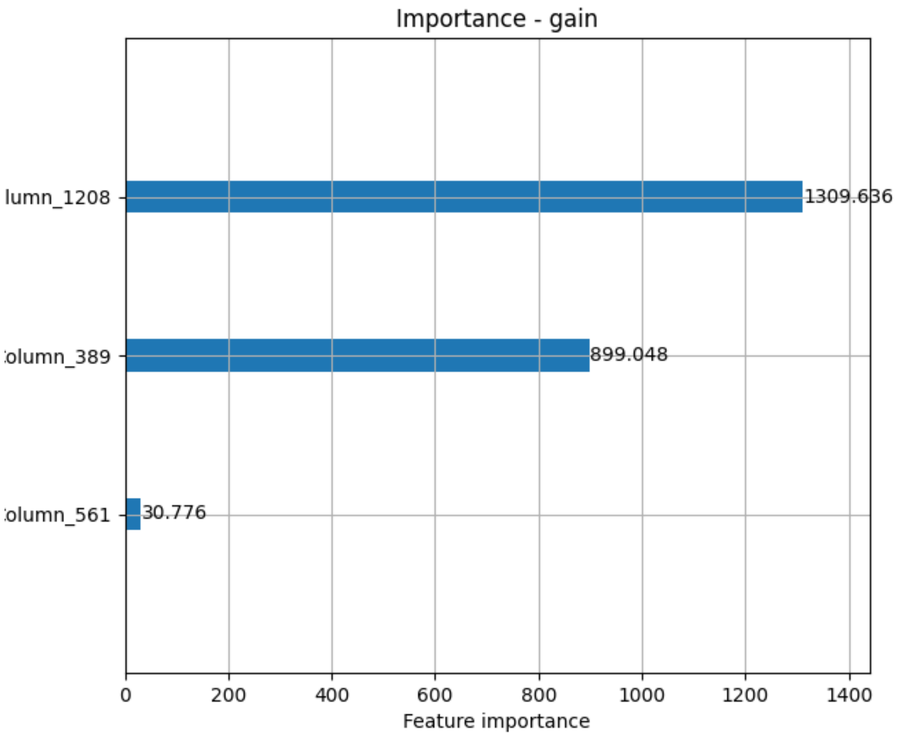

Fig. S16. Feature importance for the GLP1R allosteric ligand data set shown by the feature importance gain method in LightGBM. Here, the most important feature (depicted as column_1208) corresponds to the fingerprint bit referring to the first structural fragment shown in Figure 2, while the second structural fragment shown in Figure 2 corresponds to column_389.

Table S1. A limited validation GPCR-like data set used for comparison with other web services for target prediction.

ChEMBL ID[1]	SMILES	Known GPCR target[2]	Target selectivity[3]
CHEMBL4162364	COc1nc(F)c(F)nc1NS(=O)(=O)c1cccc(Cl)c1Cl	CCR4	selective
CHEMBL3770912	CC(C)(C)N1CCN[C@@H](C(=O)N2CCN(C(=O)Nc3nc(C(F)(F)F)c(Cl)s3)CC2)C1	CCR2	selective
CHEMBL3236549	CC(C)c1cc(N2CC[C@@H](N)C2)nc(N)n1	VPAC1	non-selective
CHEMBL5208361	Nc1nc2c(c(=O)[nH]1)C(c1cccc(Cl)c1)CC(=O)N2	PAC1	selective
CHEMBL297868	n1(nnc2=C(N=C([C])[C]=c12)N1[C][C]C2=[C]C(=[C]C(=C12)Cl)Cl)[C]([C][C])[C][C]	CRF1R	selective
CHEMBL2205810	Cc1cnc2c(ccc3cccnc32)c1	CCR1	non-selective
CHEMBL2206397	CCc1cccc(CC)c1Cc1ncc[nH]1	PAC1	non-selective
CHEMBL4162490	CN(CCCNC(=O)c1ccc(C(F)(F)F)cc1)Cc1ccccc1	VPAC1	non-selective
CHEMBL1819083	CCC(CC)Nc1c2c(nc3c(-c4ccc(OC(F)F)cc4Cl)c(C)nn13)CCC2	CRF1R	selective
CHEMBL3787077	N[C@H](CCCCNC(=O)[C@@H]1Cc2ccccc2CN1C(=O)CCC(=O)c1ccccc1)C(=O)NCC(c1ccccc1)c1ccccc1.O=C(O)C(F)(F)F	CXCR3	selective
CHEMBL2181458	CC1(C)[C@H]2CC=C(C[N+](C)(C)Cc3ccc(-c4cccc(Cl)c4Cl)cc3)[C@@H]1C2.[I-]	CXCR3	selective
CHEMBL4167445	O=S(=O)(Nc1ncc(Cl)nc1OCc1cccnc1)c1cccc(Cl)c1Cl	CCR4	selective
CHEMBL481863	c1ccc2oc(Oc3ccc(OCCN4CCCCC4)cc3)nc2c1	VPAC1	non-selective
CHEMBL250747	c1(nc2[C]=C(Cl)C(=[C]c2nc1S(=O)(=O)[C])Cl)[C]([C])[C]	GLP1R	selective
CHEMBL593442	Cc1ccnc2c1ccc1c(C)ccnc12	CCR1	non-selective
CHEMBL440687	c1ccc(N2CCN(Cc3nc4ccccc4[nH]3)CC2)nc1	VPAC1	non-selective
CHEMBL3480577	[C]C1=[C]C2=C([C]=C(N=C2[C]=[C]1)[C]N[C][C][C]OC1=[C][C]=[C][C]=[C]1)O	SCTR	non-selective
CHEMBL4067429	CC1=CCC[C@H]1NC(=O)Nc1ccc(Cl)c(S(=O)(=O)[C@@]2(C)CCOC2)c1O	CCR5	non-selective
CHEMBL250529	c1(nc2[C]=C(Cl)C(=[C]c2nc1S(=O)(=O)[C])Cl)NNC(=O)[C]	GLP1R	selective
CHEMBL250310	c1(nc2[C]=C(Cl)C(=[C]c2nc1S(=O)(=O)[C])Cl)[C][C][C]	GLP1R	selective
CHEMBL401292	c1(nc2=[C]C(=C(Cl)[C]=c2nc1[S@](=O)[C])Cl)[C]([C])[C]	GLP1R	selective
CHEMBL250112	c1(nc2[C]=C(Cl)C(=[C]c2nc1N[C]1[C][C][C]C2=C1[C]=[C][C]=[C]2)Cl)S(=O)(=O)[C]	GLP1R	selective
CHEMBL484158	CCC(CC)Nc1nc(C)nc2c(-c3cc(F)c(OC)cc3Cl)c(C)nn12	CRF1R	non-selective
CHEMBL4166905	CN(CCNC(=O)c1ccc([N+](=O)[O-])cc1)Cc1ccccc1	VPAC1	non-selective

CHEMBL399713	c1(nc2=[C]C(=C(Cl)[C]=c2nc1S(=O)(=O)[C])Cl)N[C]([C])[C][C]	GLP1R	selective
CHEMBL3986225	O=C(N[C@H]1c2ccccc2C[C@H]1O)[C@H](Cc1ccccc1)C[C@H](O)CNS(=O)(=O)c1ccc(Cl)cc1	VPAC2	selective
CHEMBL5179705	COc1cccc(C2CC(=O)Nc3nc(N)[nH]c(=O)c32)c1	PAC1	selective
CHEMBL153038	CC(C)c1ccc(Cn2ccc3c(/C=N/NC(=O)c4ccc(O)c(Cl)c4)cccc32)cc1	PAC1	non-selective
CHEMBL400500	c1(nc2=[C]C(=C(Cl)[C]=c2nc1N1[C][C]C(=O)N1)Cl)S(=O)(=O)[C]	GLP1R	selective
CHEMBL249091	c1(nc2=[C]C(=C(Cl)[C]=c2nc1S(=O)(=O)[C])Cl)N[C]([C])[C]	GLP1R	selective
CHEMBL45187	C1(=[C]C(=Nc2c(c([C])nn12)C1=C([C]=C([C]=[C]1)O[C])[C])[C])N[C]([C][C])[C][C]	CRF1R	selective
CHEMBL66165	Clc1ccc2c(NCCCCNc3ccnc4cc(Cl)ccc34)ccnc2c1	CCR1	non-selective
CHEMBL2181450	CC1(C)[C@@H]2CC=C(C[N+](C)(C)Cc3ccc(-c4ccccc4I)cc3)[C@H]1C2.[I-]	CXCR3	selective
CHEMBL506214	CC[C@@H](C)Nc1nc(C)nc2c(-c3c(C)cc(OC)cc3OC)c(C)nn12	CRF1R	non-selective
CHEMBL3941654	COc1ccc(S(=O)(=O)NC[C@@H](O)C[C@@H](Cc2ccccc2)C(=O)N[C@H]2c3ccccc3C[C@H]2O)cc1	VPAC2	selective
CHEMBL398714	c1(nc2[C]=C(Cl)C(=[C]c2nc1S(=O)(=O)[C])Cl)NC([C])([C])[C]	GLP1R	selective
CHEMBL99384	Cc1cnc2c(ccc3c(C)c(C)cnc32)c1C	CCR1	non-selective
CHEMBL18143	CCCCC(=O)O[C@H]1CCn2c1nc1c2C(=O)C(=C(NC(=O)C)C1=N)C	CCR2	non-selective
CHEMBL4519152	CC(=O)C1=C(O)C(=O)N(c2ccc(Cl)cc2F)[C@@H]1C1CCCCC1	CCR2	non-selective
CHEMBL2205805	Clc1cc(c2ccccn2)nc(c2ccccn2)c1	CCR1	non-selective
CHEMBL6273	CN1CCN(c2c(F)cc3c(=O)c(C(=O)O)cn(CCF)c3c2F)CC1	VPAC1	non-selective
CHEMBL316589	Cc1ccnc2c1ccc1cccnc21	CCR1	non-selective

[1]This ChEMBL data set was **manually curated** and labeled (selective/non-selective). 'Non-selective' means here that a compound is assigned to a few target proteins in ChEMBL.
[2]Here, in the case of non-selective ligands, only one GPCR was provided based on the assignment in source ChEMBL data sets for each GPCR target implemented in GPCRVS.
[3]Selectivity according to the ChEMBL-based target assignment.

Table S2. A comparison of GPCRVS with SwissTargetPrediction results for target and/or target class prediction – a GPCR-like data set, percentage of compounds assigned as true positives (rate of correct predictions of target or target class assignment).

Web service	TP – target prediction [%]	TP – target class prediction* [%]
GPCRVS – all three predictors	0.0	54.8**
**GPCRVS – at least 2 predictors**	**19.0 (14.3 for ML predictors only)**	**83.3** (57.1 for ML predictors only)**
**GPCRVS – at least 1 predictor**	**52.4**	**97.6****
GPCRVS – TF	28.6	64.3**
GPCRVS – LightGBM	26.2	76.2**
GPCRVS – Autodock Vina	16.7	95.2**
**SwissTargetPrediction**	**31.0**	**69.0**

* Here, target class prediction means either a correct prediction that a compound is a GPCR-targeting compound or a wrong prediction that a compound interacts with any other, non-GPCR-like drug target.
** Here, because currently only two GPCR classes are included, which makes the prediction easier, only the 1st target/target class assignment was considered for GPCRVS instead of the top three predictions like for other web services.

Table S3. A comparison of GPCRVS with SwissTargetPrediction results for target and/or target class prediction – a GPCR-like data set including only selective ligands, percentage of compounds assigned as true positives (rate of correct predictions of target or target class assignment).

Web service	TP – target prediction [%]	TP – target class prediction* [%]
GPCRVS – all three predictors	0.0	57.1**
**GPCRVS – at least 2 predictors**	**33.3 (28.6 for ML predictors only)**	**85.7** (61.9 for ML predictors only)**
**GPCRVS – at least 1 predictor**	**71.4**	**100.0****
GPCRVS – TF	38.1	61.9**
GPCRVS – LightGBM	47.6	85.7**
GPCRVS – Autodock Vina	19.0	95.2**
**SwissTargetPrediction**	**28.6**	**66.7**

* Here, target class prediction means either a correct prediction that a compound is a GPCR-targeting compound or a wrong prediction that a compound interacts with any other, non-GPCR-like drug target.
** Here, only the 1st target/target class assignment was considered for GPCRVS instead of the top three predictions like for other, multi-purpose (many drug classes) web services.

Table S4. Results for patent ligands of CCR1 (inhibitors), not included in SwissTargetPrediction or GPCRVS data sets. Correct target or target class assignment is indicated with 1, while misprediction with 0. If no prediction was provided '-' sign was included. Only one, top-scored prediction was considered for both, SwissTargetPrediction and GPCRVS.

TARGET CLASS PREDICTION							
		GPCRVS					
		orthosteric			allosteric		
Compounds	SwissTargetPrediction	TF	LIGHTGBM	VINA	TF	LIGHTGBM	VINA
US-11759454-B2_claim13_1	0	0	1	1	0	1	0
US-11759454-B2_claim13_2	1	1	1	0	1	0	0
US-11759454-B2_claim13_3	1	1	0	1	1	0	1
US-11759454-B2_claim13_5	1	1	0	1	1	0	0
US-11759454-B2_claim13_6	0	1	0	1	1	0	0
US-11759454-B2_claim13_7	0	1	0	1	1	0	1
US-11759454-B2_claim13_8	0	1	0	0	1	0	0
US-11759454-B2_claim13_9	0	1	1	1	1	1	0
US-11759454-B2_claim13_14	0	1	0	1	1	0	0
US-11759454-B2_claim14_1	0	1	0	1	1	0	1
US-11759454-B2_claim14_3	0	1	0	0	1	0	0
US-11759454-B2_claim14_4	0	1	0	1	1	0	0
US-11759454-B2_claim15	0	1	0	0	1	0	0

TARGET PREDICTION							
		GPCRVS					
		orthosteric			allosteric		
Compound	SwissTargetPrediction	TF	LIGHTGBM	VINA	TF	LIGHTGBM	VINA
US-11759454-B2_claim13_1	0	1	0	0	1	0	0
US-11759454-B2_claim13_2	0	0	0	0	0	0	0
US-11759454-B2_claim13_3	0	0	0	0	0	0	0
US-11759454-B2_claim13_5	0	0	0	0	0	0	0
US-11759454-B2_claim13_6	0	0	0	0	0	0	0
US-11759454-B2_claim13_7	0	0	0	0	0	0	0
US-11759454-B2_claim13_8	0	0	0	0	0	0	0
US-11759454-B2_claim13_9	0	0	0	0	0	0	0
US-11759454-B2_claim13_14	0	0	0	0	0	0	0
US-11759454-B2_claim14_1	0	0	0	0	0	0	0
US-11759454-B2_claim14_3	0	0	0	0	0	0	0
US-11759454-B2_claim14_4	0	0	0	0	0	0	0
US-11759454-B2_claim15	0	0	0	0	0	0	0

Table S5. Results for patent ligands of CCR2 (inhibitors), not included in SwissTargetPrediction or GPCRVS data sets.

TARGET CLASS PREDICTION							
		GPCRVS					
		orthosteric			allosteric		
Compound	SwissTargetPrediction	TF	LIGHTGBM	VINA	TF	LIGHTGBM	VINA
US10253087B2_1	0	0	0	1	0	0	1
US10253087B2_2	0	0	0	0	0	0	1
US10253087B2_3	1	1	0	0	1	0	0

US10253087B2_4	0	0	0	0	0	0	1
CA2985194C_1	1	1	0	0	1	0	1
CA2985194C_2	1	1	0	0	1	0	0
CA2985194C_3	0	0	0	0	1	0	0
CA2985194C_4	1	0	0	0	0	0	1
CA2985194C_5	1	0	0	0	0	0	0
CA2985194C_6	1	1	0	0	1	0	0
CA2985194C_7	1	1	0	1	1	0	0
CA2985194C_9	0	0	0	0	1	0	1
CA2985194C_10	0	1	0	0	1	0	0
CA2985194C_11	1	0	0	0	0	0	0
CA2985194C_12	0	1	0	1	1	0	0
CA2985194C_14	1	1	0	0	1	0	0
CA2985194C_15	1	1	0	0	1	0	0
CA2985194C_16	1	0	0	0	1	0	0
CA2985194C_17	–	1	0	1	1	0	0
CA2985194C_18	1	0	0	0	0	0	0
CA2985194C_19	1	1	0	0	1	0	0
CA2985194C_20	–	0	0	1	0	0	0
CA2985194C_21	1	0	0	0	0	0	0
CA2985194C_22	1	1	0	0	1	0	0
CA2985194C_23	1	1	0	0	1	0	0
CA2985194C_24	–	0	0	0	0	0	0
CA2985194C_25	–	1	0	0	1	0	0
CA2985194C_26	0	1	0	1	1	0	0
CA2985194C_27	1	1	0	0	1	0	1
CA2985194C_28	1	1	0	0	1	0	0

TARGET PREDICTION							
		GPCRVS					
		orthosteric			allosteric		
Compound	SwissTargetPrediction	TF	LIGHTGBM	VINA	TF	LIGHTGBM	VINA
US10253087B2_1	0	0	0	0	0	0	0
US10253087B2_2	0	0	0	0	0	0	0
US10253087B2_3	0	0	0	0	0	0	0
US10253087B2_4	0	0	0	0	0	0	0
CA2985194C_1	1	1	0	0	1	0	1
CA2985194C_2	1	0	0	0	0	0	0
CA2985194C_3	0	0	0	0	1	0	0
CA2985194C_4	0	0	0	0	0	0	0
CA2985194C_5	0	0	0	0	0	0	0
CA2985194C_6	1	0	0	0	0	0	0
CA2985194C_7	1	0	0	0	0	0	0
CA2985194C_9	0	0	0	0	0	0	0
CA2985194C_10	0	0	0	0	0	0	0
CA2985194C_11	1	1	0	0	1	0	0
CA2985194C_12	0	0	0	0	0	0	0
CA2985194C_14	1	1	0	0	1	0	0
CA2985194C_15	1	0	0	0	0	0	0
CA2985194C_16	1	1	0	0	1	0	0
CA2985194C_17	–	1	0	0	1	0	0
CA2985194C_18	1	0	0	0	1	0	0

CA2985194C_19	1	0	0	0	0	0	0
CA2985194C_20	—	1	0	0	1	0	0
CA2985194C_21	1	1	0	0	1	0	0
CA2985194C_22	1	0	0	0	0	0	0
CA2985194C_23	1	0	0	0	0	0	0
CA2985194C_24	—	0	0	0	1	0	0
CA2985194C_25	—	1	0	0	1	0	0
CA2985194C_26	0	0	0	0	0	0	0
CA2985194C_27	1	1	0	0	1	0	0
CA2985194C_28	1	0	0	0	0	0	0

Table S6. Results for patent ligands of CCR6 (inhibitors), not included in SwissTargetPrediction or GPCRVS data sets.

TARGET CLASS PREDICTION							
		GPCRVS					
		orthosteric			allosteric		
Compound	SwissTargetPrediction	TF	LIGHTGBM	VINA	TF	LIGHTGBM	VINA
US10786494B2_1	0	0	0	0	0	0	0
US10786494B2_2	1	0	0	1	0	0	1
US10786494B2_3	0	0	0	0	0	0	0
US10786494B2_4	1	0	0	0	0	0	0
US10786494B2_5	1	0	1	0	0	1	1
US10786494B2_6	0	0	1	0	0	1	0
US10786494B2_7	0	0	0	0	0	0	1
US10786494B2_8	0	0	0	0	0	0	1
US10786494B2_9	0	0	0	0	0	1	1
US10786494B2_10	0	0	1	0	0	1	1
US10786494B2_11	0	0	1	0	0	1	1
US10786494B2_12	1	0	1	0	0	1	1
US10786494B2_13	1	0	1	0	0	1	1

TARGET PREDICTION							
		GPCRVS					
		orthosteric			allosteric		
Compound	SwissTargetPrediction	TF	LIGHTGBM	VINA	TF	LIGHTGBM	VINA
US10786494B2_1	0	0	0	0	0	0	0
US10786494B2_2	0	0	0	0	0	0	0
US10786494B2_3	0	0	0	0	0	0	0
US10786494B2_4	0	0	0	0	0	0	0
US10786494B2_5	0	0	0	0	0	0	0
US10786494B2_6	0	0	0	0	0	0	0
US10786494B2_7	0	0	0	0	0	0	0
US10786494B2_8	0	0	0	0	0	0	0
US10786494B2_9	0	0	0	0	0	0	0
US10786494B2_10	0	0	0	0	0	0	0
US10786494B2_11	0	0	0	0	0	0	0
US10786494B2_12	0	0	0	0	0	0	0
US10786494B2_13	0	0	0	0	0	0	0

Table S7. Results for patent ligands of CXCR2 (inhibitors), not included in SwissTargetPrediction or GPCRVS data sets.

TARGET CLASS PREDICTION							
		GPCRVS					
		orthosteric			allosteric		
Compound	SwissTargetPrediction	TF	LIGHTGBM	VINA	TF	LIGHTGBM	VINA
US11945805B2_1	0	0	0	1	0	0	1
US11945805B2_2	0	1	0	0	1	0	1
US11945805B2_3	0	1	1	0	1	1	1
US11945805B2_4	0	1	0	0	1	0	0
US11945805B2_5	0	0	1	0	0	1	0
US11945805B2_6	–	0	0	1	0	0	0
US11945805B2_7	–	0	0	0	0	0	0
US11945805B2_8	0	0	0	1	0	1	0
US11945805B2_9	0	1	1	1	1	0	0
US11945805B2_10	–	0	1	0	0	1	0
US9115087B2_1	0	0	1	1	0	1	0
US9115087B2_3	0	0	1	1	0	1	0
US9115087B2_4	0	0	1	1	0	1	0
US9115087B2_5	0	1	1	1	1	1	1
US9115087B2_6	0	0	1	0	0	1	0
US9115087B2_7	0	0	1	1	0	1	0
US9115087B2_8	0	0	1	0	0	1	0
US9115087B2_9	1	0	1	0	0	1	0
US9115087B2_10	0	1	1	1	1	1	0
US9115087B2_11	0	1	1	1	1	1	0

TARGET PREDICTION							
		GPCRVS					
		orthosteric			allosteric		
Compound	SwissTargetPrediction	TF	LIGHTGBM	VINA	TF	LIGHTGBM	VINA
US11945805B2_1	0	0	0	1	0	0	0
US11945805B2_2	0	0	0	0	0	0	0
US11945805B2_3	0	0	0	0	0	0	0
US11945805B2_4	0	0	0	0	0	0	0
US11945805B2_5	0	0	0	0	0	0	0
US11945805B2_6	–	0	0	0	0	0	0
US11945805B2_7	–	0	0	0	0	0	0
US11945805B2_8	0	0	1	1	0	1	0
US11945805B2_9	0	1	1	0	1	1	0
US11945805B2_10	–	0	0	0	0	0	1
US9115087B2_1	0	0	1	0	0	1	0
US9115087B2_3	0	0	1	0	1	1	0
US9115087B2_4	0	0	1	0	0	1	0
US9115087B2_5	0	1	1	0	1	1	0
US9115087B2_6	0	0	1	0	1	1	0
US9115087B2_7	0	1	1	0	1	1	0
US9115087B2_8	0	1	1	0	1	1	0
US9115087B2_9	0	1	1	0	1	0	0
US9115087B2_10	0	1	1	0	1	1	0
US9115087B2_11	0	1	1	0	1	1	0

Table S8. Results for patent ligands of CXCR3 (inhibitors), not included in SwissTargetPrediction or GPCRVS data sets.

TARGET CLASS PREDICTION							
		GPCRVS					
		orthosteric			allosteric		
Compound	SwissTargetPrediction	TF	LIGHTGBM	VINA	TF	LIGHTGBM	VINA
EP1853587B1_1	1	0	1	0	1	1	0
EP1853587B1_2	1	0	1	0	1	1	0
EP1853587B1_3	1	1	1	0	1	1	1
EP1853587B1_4	1	1	1	1	1	1	1
EP1853587B1_5	1	1	1	1	1	1	0
EP1853587B1_6	1	1	1	0	1	1	0
EP1853587B1_7	1	1	1	0	1	1	1
EP1853587B1_8	1	0	1	1	1	1	1
EP1853587B1_9	1	0	1	1	0	1	1
EP1853587B1_10	1	0	1	0	0	1	0
EP1858895B1_1	1	0	1	0	0	1	0
EP1858895B1_2	0	0	1	1	0	1	1
EP1858895B1_3	0	0	1	0	0	1	0
EP1858895B1_4	0	0	1	1	0	1	1
EP1858895B1_5	0	0	1	0	0	1	0
EP1858895B1_6	1	0	1	1	0	1	0
EP1858895B1_7	1	0	1	1	0	1	1
EP1858895B1_8	1	0	1	1	0	1	1
EP1858895B1_9	1	0	1	1	0	0	0
EP1858895B1_10	0	0	1	0	0	1	1

TARGET PREDICTION							
		GPCRVS					
		orthosteric			allosteric		
Compound	SwissTargetPrediction	TF	LIGHTGBM	VINA	TF	LIGHTGBM	VINA
EP1853587B1_1	1	1	1	0	1	1	0
EP1853587B1_2	1	1	1	0	1	1	0
EP1853587B1_3	1	1	1	0	1	1	1
EP1853587B1_4	1	1	1	0	1	1	0
EP1853587B1_5	1	1	1	1	1	1	0
EP1853587B1_6	1	1	1	0	1	1	0
EP1853587B1_7	1	1	1	0	1	1	0
EP1853587B1_8	1	1	1	0	1	1	1
EP1853587B1_9	1	0	1	0	0	1	1
EP1853587B1_10	1	0	1	0	0	1	0
EP1858895B1_1	1	0	1	0	1	1	0
EP1858895B1_2	0	0	1	1	0	1	1
EP1858895B1_3	0	0	1	0	1	1	0
EP1858895B1_4	0	0	1	1	1	1	1
EP1858895B1_5	0	0	1	0	1	1	0
EP1858895B1_6	0	0	1	0	0	1	0
EP1858895B1_7	0	0	1	0	0	1	0
EP1858895B1_8	0	0	1	0	0	1	0
EP1858895B1_9	0	0	1	0	0	1	0
EP1858895B1_10	0	0	1	0	1	1	0

Table S9. Results for patent ligands of CRF1R (inhibitors), not included in SwissTargetPrediction or GPCRVS data sets.

TARGET CLASS PREDICTION							
		GPCRVS					
		orthosteric			allosteric		
Compound	SwissTargetPrediction	TF	LIGHTGBM	VINA	TF	LIGHTGBM	VINA
CN101142217B_1	0	1	1	0	1	1	1
CN101142217B_2	1	1	1	1	1	1	0
CN101142217B_3	1	1	1	0	1	1	0
CN101142217B_4	1	1	1	0	1	1	0
CN101142217B_5	1	1	1	0	1	1	0
CN101142217B_6	1	1	1	0	1	0	0
CN101142217B_7	1	0	1	1	0	1	0
CN101142217B_8	1	1	1	0	1	1	0
CN101142217B_9	0	0	1	0	0	1	0
CN101142217B_10	1	0	1	0	0	1	1
CN101142217B_11	0	0	1	0	0	1	0
CN101142217B_12	0	0	0	1	0	1	1
CN101142217B_13	0	0	1	0	0	1	0
CN101142217B_14	0	0	1	0	0	0	0

TARGET PREDICTION							
		GPCRVS					
		orthosteric			allosteric		
Compound	SwissTargetPrediction	TF	LIGHTGBM	VINA	TF	LIGHTGBM	VINA
CN101142217B_1	0	1	1	0	0	0	0
CN101142217B_2	1	1	1	0	1	0	0
CN101142217B_3	1	1	1	0	1	0	0
CN101142217B_4	1	1	1	0	1	0	0
CN101142217B_5	1	1	1	0	0	0	0
CN101142217B_6	1	1	1	0	0	0	0
CN101142217B_7	1	0	1	0	0	0	0
CN101142217B_8	1	1	1	0	1	0	0
CN101142217B_9	0	1	1	0	1	0	0
CN101142217B_10	1	1	1	0	1	0	0
CN101142217B_11	0	1	1	0	1	0	0
CN101142217B_12	0	0	0	0	0	0	0
CN101142217B_13	0	0	1	0	0	0	0
CN101142217B_14	0	0	1	0	0	0	0

Table S10. Results for patent ligands of GCGR (agonists), not included in SwissTargetPrediction or GPCRVS data sets.

TARGET CLASS PREDICTION							
		GPCRVS					
		orthosteric			allosteric		
Compound	SwissTargetPrediction	TF	LIGHTGBM	VINA	TF	LIGHTGBM	VINA
EP3209682B1_1	0	0	1	1	0	1	1
EP3209682B1_2	0	0	1	0	0	1	1
EP3209682B1_3	0	0	1	0	0	1	1
EP3209682B1_4	0	0	1	1	0	1	1
EP3209682B1_5	0	0	1	1	0	1	0
US10479819B2_1	0	0	1	1	0	1	1
US11542313B2_1	0	1	1	0	1	1	1
RU2760007C2_1	0	1	1	1	1	1	1
RU2760007C2_2	0	1	1	1	1	1	1
RU2760007C2_3	0	0	1	1	0	1	1
RU2760007C2_4	0	0	1	1	0	1	1
RU2760007C2_5	0	0	1	1	0	1	0

TARGET PREDICTION							
		GPCRVS					
		orthosteric			allosteric		
Compound	SwissTargetPrediction	TF	LIGHTGBM	VINA	TF	LIGHTGBM	VINA
EP3209682B1_1	0	0	0	0	0	1	1
EP3209682B1_2	0	0	0	0	0	1	0
EP3209682B1_3	0	0	0	0	0	1	0
EP3209682B1_4	0	0	0	0	0	1	0
EP3209682B1_5	0	0	0	0	0	1	0
US10479819B2_1	0	0	0	0	0	0	0
US11542313B2_1	0	0	0	0	0	0	0
RU2760007C2_1	0	0	0	1	0	1	0
RU2760007C2_2	0	0	0	1	0	1	1
RU2760007C2_3	0	0	0	0	0	1	0
RU2760007C2_4	0	0	0	1	0	1	1
RU2760007C2_5	0	0	0	1	0	1	0

Table S11. Results for patent ligands of GLP1R (agonists), not included in SwissTargetPrediction or GPCRVS data sets.

TARGET CLASS PREDICTION							
		GPCRVS					
		orthosteric			allosteric		
Compound	SwissTargetPrediction	TF	LIGHTGBM	VINA	TF	LIGHTGBM	VINA
US10954221B2_1	0	1	0	0	1	1	0
US10954221B2_2	0	1	0	1	1	1	0
TWI819518B_1	0	1	1	0	0	1	0
TWI819518B_2	0	1	1	1	1	1	1
TWI819518B_3	0	1	1	1	1	1	0
TWI819518B_4	0	1	1	0	1	1	1
TWI819518B_5	0	0	1	1	0	1	1
TWI819518B_6	0	1	1	0	0	1	0
TWI819518B_7	0	1	1	0	1	1	0
TWI819518B_8	0	1	1	1	1	1	0
TWI819518B_9	0	1	1	0	1	1	0
TWI819518B_10	0	1	1	1	1	1	0
US11542313B2_1	0	1	1	0	1	1	1
EP3209682B1_1	0	0	1	1	0	1	1
EP3209682B1_2	0	0	1	0	0	1	1
EP3209682B1_3	0	0	1	0	0	1	1
EP3209682B1_4	0	0	1	1	0	1	1
EP3209682B1_5	0	0	1	1	0	1	0
US10479819B2_1	0	0	1	1	0	1	1

TARGET PREDICTION							
		GPCRVS					
		orthosteric			allosteric		
Compound	SwissTargetPrediction	TF	LIGHTGBM	VINA	TF	LIGHTGBM	VINA
US10954221B2_1	0	0	0	0	0	0	0
US10954221B2_2	0	0	0	0	0	0	0
TWI819518B_1	0	0	1	0	0	0	0
TWI819518B_2	0	0	1	0	0	0	0
TWI819518B_3	0	0	1	0	0	0	0
TWI819518B_4	0	0	1	0	0	0	0
TWI819518B_5	0	0	1	0	0	0	0
TWI819518B_6	0	0	0	0	0	0	0
TWI819518B_7	0	0	0	0	0	0	0
TWI819518B_8	0	0	1	0	0	0	0
TWI819518B_9	0	0	0	0	0	0	0
TWI819518B_10	0	0	1	0	0	0	0
US11542313B2_1	0	0	1	0	0	0	0
EP3209682B1_1	0	0	1	0	0	0	0
EP3209682B1_2	0	0	1	0	0	0	0
EP3209682B1_3	0	0	1	0	0	0	0
EP3209682B1_4	0	0	1	0	0	0	0
EP3209682B1_5	0	0	1	0	0	0	0
US10479819B2_1	0	0	1	0	0	0	0

Table S12. Results for patent ligands of GIPR (agonists), not included in SwissTargetPrediction or GPCRVS data sets.

TARGET CLASS PREDICTION							
		GPCRVS					
		orthosteric			allosteric		
Compound	SwissTargetPrediction	TF	LIGHTGBM	VINA	TF	LIGHTGBM	VINA
US11542313B2_1	0	1	1	0	1	1	1
US11897926B2_1	0	1	1	1	1	1	1
US11897926B2_2	0	1	1	1	1	1	1
US11897926B2_3	0	1	1	1	1	1	1

TARGET PREDICTION							
		GPCRVS					
		orthosteric			allosteric		
Compound	SwissTargetPrediction	TF	LIGHTGBM	VINA	TF	LIGHTGBM	VINA
US11542313B2_1	0	0	1	0	0	0	1
US11897926B2_1	0	0	1	1	0	0	1
US11897926B2_2	0	0	1	1	0	0	1
US11897926B2_3	0	0	1	0	0	0	1

Table S13. Results for patent ligands of VPAC2 (agonists), not included in SwissTargetPrediction or GPCRVS data sets.

TARGET CLASS PREDICTION							
		GPCRVS					
		orthosteric			allosteric		
Compound	SwissTargetPrediction	TF	LIGHTGBM	VINA	TF	LIGHTGBM	VINA
AU2016200897B2_1	0	0	1	0	0	1	1
AU2016200897B2_2	0	0	1	1	0	1	1

TARGET PREDICTION							
		GPCRVS					
		orthosteric			allosteric		
Compound	SwissTargetPrediction	TF	LIGHTGBM	VINA	TF	LIGHTGBM	VINA
AU2016200897B2_1	0	0	0	0	0	0	0
AU2016200897B2_2	0	0	0	0	0	0	0

Table S14. Training data sets used to generate TF predictors for each receptor – orthosteric ligands.

**Receptor**	**Training set**
AM1-active	orthosteric class B
AM2-active	orthosteric class B
AMY1-active	orthosteric class B
AMY2-active	orthosteric class B
AMY3-active	orthosteric class B
CCR1-active	CCR1-inactive
CCR1-inactive	CCR1-inactive
CCR2-active	CCR2-inactive
CCR2-inactive	CCR2-inactive
CCR3-active	CCR3-inactive
CCR3-inactive	CCR3-inactive
CCR4-active	CCR4-inactive
CCR4-inactive	CCR4-inactive
CCR5-active	CCR5-inactive
CCR5-inactive	CCR5-inactive
CCR6-active	CCR6-inactive
CCR6-inactive	CCR6-inactive
CGRPR-active	orthosteric class B
CALCRL-inactive	orthosteric CALCRL-inactive
CRF1R-active	CRF1R-inactive
CRF1R-inactive	CRF1R-inactive
CRF2R-active	CRF1R-inactive
CXCR1-active	CXCR1-inactive
CXCR1-inactive	CXCR1-inactive
CXCR2-active	CXCR2-inactive
CXCR2-inactive	CXCR2-inactive
CXCR3-active	CXCR3-inactive
CXCR3-inactive	CXCR3-inactive
GCGR-active	orthosteric GCGR-inactive
GCGR-inactive	orthosteric GCGR-inactive
GHRHR-active	orthosteric class B
GIPR-active	orthosteric GLP1R-inactive
GIPR-inactive	orthosteric GLP1R-inactive
GLP1R-active	orthosteric GLP1R-inactive
GLP1R-inactive	orthosteric GLP1R-inactive
PAC1-active	orthosteric class B
PAC1-inactive	orthosteric class B
PTHR1-active	orthosteric class B
PTHR2-active	orthosteric class B
SCTR-active	orthosteric class B
VPAC1-active	orthosteric class B
VPAC1-inactive	orthosteric class B
VPAC2-active	orthosteric class B
VPAC2-inactive	VPAC2-inactive

Table S15. Training data sets used to generate TF predictors for each receptor – allosteric ligands.

**Receptor**	**Training set**
AM1-active	allosteric class B
AM2-active	allosteric class B
AMY1-active	allosteric class B
AMY2-active	allosteric class B
AMY3-active	allosteric class B
CCR1-active	CCR1-inactive
CCR1-inactive	CCR1-inactive
CCR2-active	CCR2-inactive
CCR2-inactive	CCR2-inactive
CCR3-active	CCR3-inactive
CCR3-inactive	CCR3-inactive
CCR4-active	CCR4-inactive
CCR4-inactive	CCR4-inactive
CCR5-active	CCR5-inactive
CCR5-inactive	CCR5-inactive
CCR6-active	CCR6-inactive
CCR6-inactive	CCR6-inactive
CGRPR-active	allosteric class B
CALCRL-inactive	allosteric CALCRL-inactive
CRF1R-active	allosteric CRF1R-inactive
CRF1R-inactive	allosteric CRF1R-inactive
CRF2R-active	allosteric CRF1R-inactive
CXCR1-active	CXCR1-inactive
CXCR1-inactive	CXCR1-inactive
CXCR2-active	CXCR2-inactive
CXCR2-inactive	CXCR2-inactive
CXCR3-active	CXCR3-inactive
CXCR3-inactive	CXCR3-inactive
GCGR-active	allosteric GCGR-inactive
GCGR-inactive	allosteric GCGR-inactive
GHRHR-active	allosteric class B
GIPR-active	allosteric class B
GIPR-inactive	allosteric class B
GLP1R-active	allosteric class B
GLP1R-inactive	allosteric class B
PAC1-active	allosteric class B
PAC1-inactive	allosteric class B
PTHR1-active	allosteric class B
PTHR2-active	allosteric class B
SCTR-active	allosteric class B
VPAC1-active	allosteric class B
VPAC1-inactive	allosteric class B
VPAC2-active	allosteric class B
VPAC2-inactive	VPAC2-inactive

Table S16. Training data sets used to generate LightGBM predictors for each receptor – orthosteric ligands.

Receptor	Training set
AM1-active	orthosteric class B
AM2-active	orthosteric class B
AMY1-active	orthosteric class B
AMY2-active	orthosteric class B
AMY3-active	orthosteric class B
CCR1-active	CCR1-inactive
CCR1-inactive	CCR1-inactive
CCR2-active	CCR2-inactive
CCR2-inactive	CCR2-inactive
CCR3-active	CCR3-inactive
CCR3-inactive	CCR3-inactive
CCR4-active	CCR4-inactive
CCR4-inactive	CCR4-inactive
CCR5-active	CCR5-inactive
CCR5-inactive	CCR5-inactive
CCR6-active	CCR6-inactive
CCR6-inactive	CCR6-inactive
CGRPR-active	orthosteric class B
CALCRL-inactive	CALCRL-inactive
CRF1R-active	orthosteric CRF1R-inactive
CRF1R-inactive	orthosteric CRF1R-inactive
CRF2R-active	orthosteric CRF1R-inactive
CXCR1-active	CXCR1-inactive
CXCR1-inactive	CXCR1-inactive
CXCR2-active	CXCR2-inactive
CXCR2-inactive	CXCR2-inactive
CXCR3-active	CXCR3-inactive
CXCR3-inactive	CXCR3-inactive
GCGR-active	orthosteric GCGR-inactive
GCGR-inactive	orthosteric GCGR-inactive
GHRHR-active	orthosteric class B
GIPR-active	orthosteric GLP1R-inactive
GIPR-inactive	orthosteric GLP1R-inactive
GLP1R-active	orthosteric GLP1R-inactive
GLP1R-inactive	orthosteric GLP1R-inactive
PAC1-active	orthosteric class B
PAC1-inactive	orthosteric class B
PTHR1-active	orthosteric class B
PTHR2-active	orthosteric class B
SCTR-active	orthosteric class B
VPAC1-active	orthosteric class B
VPAC1-inactive	orthosteric class B
VPAC2-active	orthosteric class B
VPAC2-inactive	VPAC2-inactive

Table S17. Training data sets used to generate LightGBM predictors for each receptor – allosteric ligands.

Receptor	Training set
AM1-active	allosteric class B
AM2-active	allosteric class B
AMY1-active	allosteric class B
AMY2-active	allosteric class B
AMY3-active	allosteric class B
CCR1-active	CCR1-inactive
CCR1-inactive	CCR1-inactive
CCR2-active	CCR2-inactive
CCR2-inactive	CCR2-inactive
CCR3-active	CCR3-inactive
CCR3-inactive	CCR3-inactive
CCR4-active	CCR4-inactive
CCR4-inactive	CCR4-inactive
CCR5-active	CCR5-inactive
CCR5-inactive	CCR5-inactive
CCR6-active	CCR6-inactive
CCR6-inactive	CCR6-inactive
CGRPR-active	allosteric class B
CALCRL-inactive	CALCRL-inactive
CRF1R-active	allosteric CRF1R-inactive
CRF1R-inactive	allosteric CRF1R-inactive
CRF2R-active	allosteric CRF1R-inactive
CXCR1-active	CXCR1-inactive
CXCR1-inactive	CXCR1-inactive
CXCR2-active	CXCR2-inactive
CXCR2-inactive	CXCR2-inactive
CXCR3-active	CXCR3-inactive
CXCR3-inactive	CXCR3-inactive
GCGR-active	allosteric GCGR-inactive
GCGR-inactive	allosteric GCGR-inactive
GHRHR-active	allosteric class B
GIPR-active	allosteric GLP1R-inactive
GIPR-inactive	allosteric GLP1R-inactive
GLP1R-active	allosteric GLP1R-inactive
GLP1R-inactive	allosteric GLP1R-inactive
PAC1-active	allosteric class B
PAC1-inactive	allosteric class B
PTHR1-active	allosteric class B
PTHR2-active	allosteric class B
SCTR-active	allosteric class B
VPAC1-active	allosteric class B
VPAC1-inactive	allosteric class B
VPAC2-active	allosteric class B
VPAC2-inactive	VPAC2-inactive
